# Sorting the mind: cognitive enhancement through transcutaneous auricular vagus nerve stimulation: a systematic review and meta-analysis

**DOI:** 10.1017/S0033291726105017

**Published:** 2026-06-24

**Authors:** Fangqing Liu, Yixuan Li

**Affiliations:** 1 The University of Manchester, UK; 2 School of Health in Social Sciences, The University of Edinburgh, UK

**Keywords:** cognitive enhancement, executive functions, meta-analysis, neuromodulation, taVNS, transcutaneous auricular vagus nerve stimulation, vagal neurocognitive integration model, working memory

## Abstract

Transcutaneous auricular vagus nerve stimulation (taVNS) is a noninvasive technique engaging vagal afferents that may enhance cognition, but results vary across domains and samples. Following PRISMA, seven databases (inception–October 2025) plus registries and gray literature were searched. Random-effects meta-analyses (REML; Hedges’ *g*) were complemented by Bayesian hierarchical models and sensitivity analyses. Fifty-three studies were included; 30 contributed quantitative data (>1,500 participants). taVNS was associated with improved cognitive performance overall (*g* = 0.41, 95% CI: 0.30–0.53; *I*^2^ = 51.4%). Effects were moderate for executive functions (*g* = 0.46, 95% CI: 0.27–0.65; *I*^2^ = 9.5%) and cognitive flexibility/learning (*g* = 0.53, 95% CI: 0.32–0.75; *I*^2^ = 52.9%), and small for working memory/attention (*g* = 0.19, 95% CI: 0.04–0.33; *I*^2^ = 14.9%). Social cognition/emotion regulation showed larger but imprecise effects (*k* = 3; *g* = 0.80, 95% CI: 0.07–1.52; *I*^2^ = 82.1%). Clinical samples benefited similarly (*k* = 7; *g* = 0.55, 95% CI: 0.31–0.79; *I^2^* = 29.5%), with no difference from healthy cohorts (*β* = −0.001, *p* = .994). High-intensity protocols (>1.0 mA) yielded larger effects; mode, duration, and site were not moderators. Bayesian models supported effects (*P* [*μ* > 0] ≥ 0.93). taVNS is associated with statistically significant improvements in cognitive performance, strongest for executive control and adaptive learning. We propose a Vagal Neurocognitive Integration Model linking LC-NE arousal modulation to prefrontal control. Future diagnosis-specific, adequately powered trials with multimodal neuroimaging should refine mechanisms and dose–response.

## Background

Cognition refers to the mental processes underlying perception, attention, memory, reasoning, and decision-making. These processes are fundamental to adaptive functioning, enabling the integration of sensory inputs, the regulation of arousal states, and the planning of goal-directed behavior. Cognition encompasses not only the perception and processing of external information but also the interpretation and generation of adaptive responses (Diamond, 2013). Well-developed cognitive abilities aid effective decision-making in complex environments, foster mental well-being (Gao et al., 2022), and promote social integration (Ni et al., 2024). Conversely, cognitive impairment or decline may lead to attention deficits (Allain et al., 2007), learning difficulties (Zhong et al., 2024), and emotional dysregulation (Gao et al., 2022). Therefore, researching and enhancing cognitive functions hold significant theoretical and practical importance for improving an individual’s quality of life and social adaptability.

Our exploration into enhancing cognitive functions has never ceased. Previous cognitive improvement methods primarily relied on traditional behavior therapy and educational interventions (Birtwistle, Chernikova, Wünsch, & Niklas, 2025; Gobet & Sala, 2023; Lövdén et al., 2020), aiming to elevate an individual’s cognitive abilities through repetitive practice and memory reinforcement. However, as our understanding of brain structure and function deepens, we have discovered that cognitive functions are not solely dependent on knowledge accumulation and practice but are also profoundly influenced by neurobiological factors (Diamond & Amso, 2008; Morozova et al., 2022). This has led us to gradually expand our research perspective from external environmental factors to the neural mechanisms within the brain when exploring pathways for cognitive enhancement.

The past two decades have witnessed an increasing emphasis on neuromodulation as a strategy to address core deficits in disorders that remain refractory to conventional care (see in Krishna & Fasano, 2024). Neuromodulation targets dysfunctional circuits directly via controlled electrical or magnetic stimulation, offering the prospect of more rapid, mechanistically grounded, and durable change in brain–cognition relationships (Neves, Ventura, & Madeira, 2025; Qiao et al., 2020). Among noninvasive approaches, transcranial magnetic stimulation (TMS) and transcranial direct current stimulation (tDCS) modulate neuronal activity via magnetic fields or weak electrical currents, enabling targeted, region-specific modulation that can enhance cognitive functions (Elder & Taylor, 2014; Qiu, He, Cao, & Zhang, 2023). These techniques have shown potential to improve working memory, attention, and emotion regulation (Chen, Ke, Ni, & Ming, 2024; Davidson et al., 2024; Elder & Taylor, 2014; Mattioli et al., 2024; Vega et al., 2025). Both techniques, however, have limited capacity to engage deeper limbic structures (Camacho-Conde, Del Rosario Gonzalez-Bermudez, Carretero-Rey, & Khan, 2023). At the invasive end of the spectrum, deep brain stimulation (DBS) is highly effective for treatment-resistant movement disorders and is under active study for psychiatric disorders (Hardesty & Sackeim, 2007), but it is resource-intensive and ethically challenging. Implanted vagus nerve stimulation (VNS) has demonstrated robust long-term benefits in epilepsy (Toffa et al., 2020) and depression (Milby, Halpern, & Baltuch, 2008), highlighting the therapeutic potential of vagal pathways, yet the requirement of surgical implantation and risk of complications restricts its wider use.

**Figure 1.**
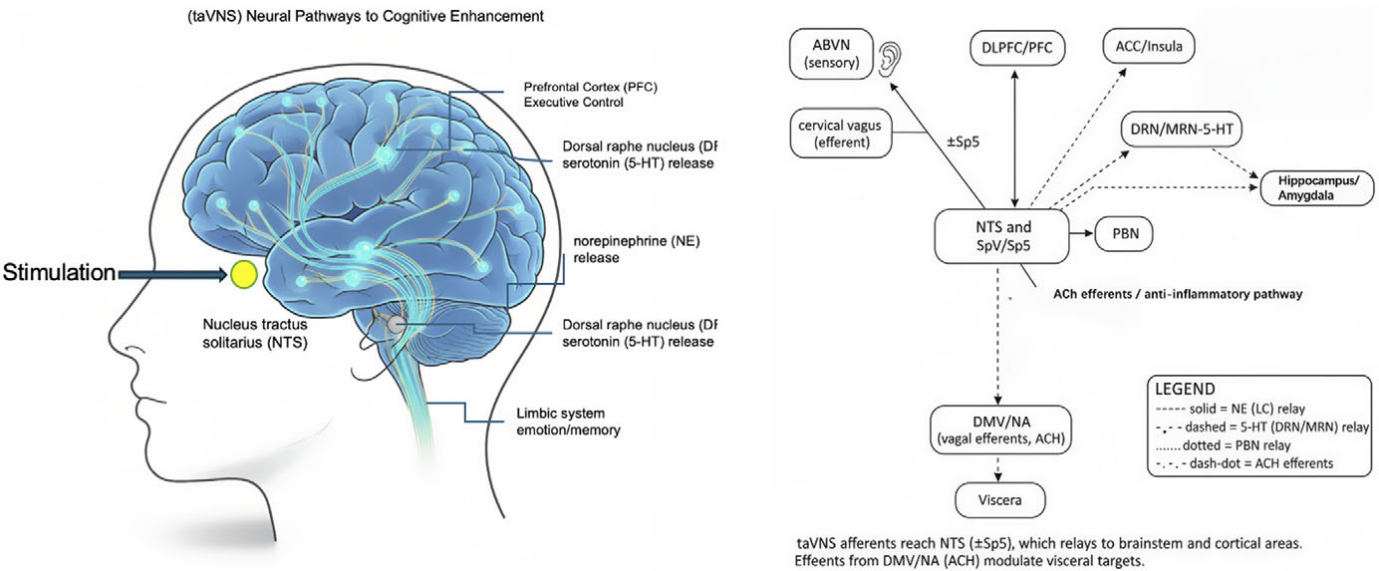
taVNS afferent pathways and modulatory/efferent effects. *Note:* Afferent inputs from the auricular branch of the vagus (ABVN) project to the nucleus tractus solitarius (NTS) (± spinal trigeminal nucleus, SpV/Sp5). NTS engages brainstem modulatory nuclei–locus coeruleus (LC; norepinephrine, NE) and dorsal/median raphe (DRN/MRN; serotonin, 5-HT), and relays (via parabrachial nucleus, PBN) to cortical/limbic targets (DLPFC/PFC, ACC/Insula, and Hippocampus/Amygdala). Vagal efferents arise from the dorsal motor nucleus/nucleus ambiguus (DMV/NA) and project to viscera (acetylcholine, ACh; anti-inflammatory pathway). Line styles: solid = NE (LC); dashed = 5-HT (DRN/MRN); dotted = PBN relay; dash-dot = ACh efferents. Figure 1. Long description.

Against this backdrop, neuromodulation has reframed a familiar question: what if we could modulate the neural circuits that regulate arousal, affect, and inhibitory control – directly and noninvasively? Transcutaneous auricular vagus nerve stimulation (taVNS) has emerged as an especially compelling alternative. By stimulating the auricular branch of the vagus nerve (ABVN) at the external ear, taVNS provides noninvasive access to vagal afferents while avoiding surgical risks (Badran et al., 2019; Butt, Albusoda, Farmer, & Aziz, 2020). These afferents terminate in the nucleus tractus solitarius (NTS), which relays to the locus coeruleus, and limbic and prefrontal regions (amygdala, hippocampus, and medial prefrontal cortex) implicated in emotion regulation, autonomic balance, and executive control (Caestecker et al., 2025). These projections enable taVNS to influence broad neuromodulatory systems, including noradrenergic, cholinergic, and serotonergic pathways, thereby exerting effects on arousal, attention, and affect regulation (Aranberri Ruiz, 2024) (see Figure 1).

### Sorting the mind: Cognitive functions in taVNS

**Figure 2.**
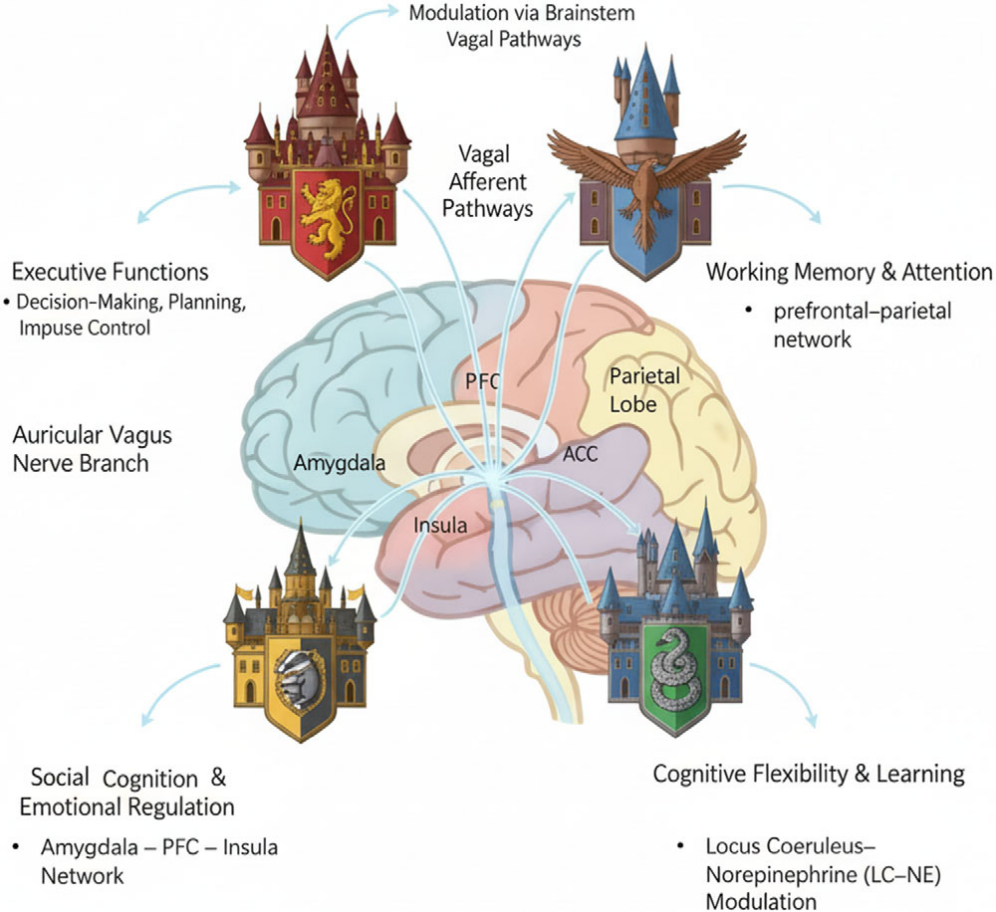
Cognitive functions and taVNS: A four-domain framework. Figure 2. long description.

An increasing number of studies are now focusing on the effects of taVNS across specific cognitive domains (see in Ridgewell et al., 2021). However, cognitive functions do not operate in isolation; they are subserved by distributed neural networks involving multiple brain regions and interconnected circuits. Modulation of one component may therefore produce cascading effects across the cognitive system. To systematically evaluate how taVNS influences cognition, we organized the literature according to four theoretically grounded domains (see Figure 2).

### Executive functions

Executive functions govern higher-order cognitive activities, such as decision-making, planning, problem-solving, and impulse control. They are not only important for individuals navigating complex environments but also represent a core capacity for flexible adaptation and adjustment to change, enabling individuals to demonstrate ‘resolve’ and ‘initiative’ when confronting intricate decisions (Diamond, 2013). Whether taVNS can enhance adaptive capacity in complex environments by stimulating the prefrontal cortex to improve impulse control and decision-making represents one of the primary foci of our current exploration (Mena-Chamorro et al., 2025; Zhang et al., 2024).

### Working memory and attention

Working memory represents the mental workspace that allows individuals to temporarily store, update, and manipulate information essential for reasoning, comprehension, and goal-directed behavior. It is not merely a passive holding system but a dynamic process that integrates incoming sensory input with existing knowledge to facilitate complex cognitive operations (Baddeley, 1992). Attention, on the other hand, functions as the selective gatekeeper of cognition, allocating limited neural resources toward task-relevant stimuli while inhibiting distractions (van Moorselaar & Slagter, 2020). From a neurocognitive perspective, these two mechanisms are deeply interwoven. The central executive component of working memory relies heavily on attentional control systems mediated by the prefrontal cortex and parietal networks (Friedman & Robbins, 2022; Funahashi, 2017). This interaction enables individuals to maintain focus under conditions of high cognitive load and to flexibly shift attention when task demands change (Egner, 2023).

### Social cognition and emotional regulation

Social cognition encompasses a constellation of mental processes that enable individuals to perceive, interpret, and predict others’ emotions, intentions, and social behaviors (Arioli, Crespi, & Canessa, 2018). It forms the cognitive foundation for empathy, moral reasoning, and adaptive interpersonal functioning (Arioli, Crespi, & Canessa, 2018). Emotional regulation, by contrast, refers to the capacity to monitor, evaluate, and modify emotional reactions to maintain psychological homeostasis and achieve social harmony (McRae & Gross, 2020). Together, these systems constitute the core of affective intelligence – an essential element of prosocial behavior and interpersonal sensitivity.

From a neurobiological perspective, both social cognition and emotional regulation are supported by overlapping neural circuits involving the prefrontal cortex, amygdala, insula, and anterior cingulate cortex (Gangopadhyay, Chawla, Dal Monte, & Chang, 2021). These regions mediate the intricate balance between emotional reactivity and cognitive control (Casey, Heller, Gee, & Cohen, 2019). taVNS, by modulating vagal afferent pathways and enhancing parasympathetic tone, may exert a dual influence: strengthening emotion recognition and improving self-regulatory capacity. Such modulation could facilitate more adaptive social responses, attenuate maladaptive emotional arousal, and enhance empathy-driven interactions.

### Cognitive flexibility and learning

Cognitive flexibility reflects an individual’s ability to disengage from habitual responses and adopt novel, contextually appropriate solutions – an essential mechanism for strategic reasoning and adaptive problem-solving (Corbo, Troisi, Marselli, & Casagrande, 2024; Ionescu, Rabaglietti, & Miu, 2024). Complementing this, learning ability represents the progressive consolidation of knowledge and experience through iterative feedback and neural plasticity, ensuring that past encounters inform future decisions (Galván, 2010). Together, these capacities underpin adaptive behavior and the ability to modify strategies in response to environmental feedback. taVNS via the locus coeruleus–norepinephrine (LC–NE) system, may strengthen the neural substrates supporting cognitive flexibility and adaptive learning. This mechanism is hypothesized to promote heightened alertness, more efficient error monitoring, and greater behavioral plasticity.

### The present study

The burgeoning interest in taVNS represents a rapidly evolving field within cognitive enhancement research. However, the existing research landscape remains fragmented, with disparate findings across domains. Previous meta-analyses of taVNS have largely examined noncognitive outcomes – insomnia (de Oliveira et al., 2025), pupil dilation (Pervaz et al., 2025), heart-rate variability (HRV) (Wolf et al., 2021), and overall safety (Kim et al., 2022). To date, only one quantitative synthesis has evaluated cognitive effects; however, it was restricted to healthy individuals and did not examine domain-specific outcomes, reporting a small but significant pooled effect (*g* = 0.21) across 19 studies (Ridgewell et al., 2021). However, emerging studies conducted over the past 5 years in various populations have yielded conflicting findings. Some studies included in previous meta-analyses have been retracted due to problematic data reporting, which also calls into question the robustness of earlier findings. This highlights the need for a more updated, comprehensive meta-analytic synthesis that consolidates results across diverse studies.

### Research objectives

The research objective (RO)s of this study is therefore threefold:

RO1: Assess the overall effect of taVNS on cognitive functions and systematically evaluate the impact of taVNS on various cognitive domains.

RO2: Investigate the impact of taVNS on clinical populations to assess how taVNS affects individuals with cognitive impairments related to various conditions.

RO3: Examine moderator variables and heterogeneity in effect sizes to explore the role of different moderators (e.g. stimulation parameters like frequency, intensity, and duration) on the effectiveness of taVNS.

## Methodology

### Protocol and registration

This systematic review and meta-analysis followed the Preferred Reporting Items for Systematic Reviews and Meta-Analyses (PRISMA) guidelines and is prospectively registered in the PROSPERO International Prospective Register of Systematic Reviews (ID: CRD 420251166774). The completed PRISMA checklist is provided in Supplementary Appendix A.

### Search strategy

We developed a comprehensive search strategy with a specialist librarian to cover biomedical, psychological, and interdisciplinary literature (see Supplementary Appendix B, Supplementary Tables B1–B2). Seven databases were searched from inception to October 2025: PubMed, MEDLINE, EMBASE, PsycINFO, Web of Science Core Collection, Cochrane CENTRAL, and Scopus. To minimize publication bias, we also searched ClinicalTrials.gov and the WHO ICTRP. Hand-searching included reference lists of eligible full texts and relevant reviews/meta-analyses. Gray literature was screened via ProQuest Dissertations and Theses Global, and reports from scientific meetings and other unpublished sources were considered where methods and outcomes were adequately reported. No language or year restrictions were applied.

### Study selection and data extraction

Study selection was performed independently by two reviewers. Inter-rater agreement was excellent (abstract screening: Cohen’s *κ* = 0.96; full-text screening: Cohen’s *κ* = 0.92). Inclusion/exclusion criteria and types of data collected from each article presented in this analysis can be found in Supplementary Appendix B, Supplementary Tables B3 and B4; the data extraction results can be found in Supplementary Appendix C.

### Statistical analysis

All quantitative syntheses were conducted in R (v 4.3.1) using the *metafor* (Viechtbauer, 2010) and *brm*s (Bürkner, 2017) packages. Standardized mean differences were expressed as Hedges’ *g.* Compared to Cohen’s *d*, Hedges’ *g* corrects for small-sample bias with a correction factor, making it more suitable for the small-to-medium sample studies prevalent in this field. For the primary analyses, a random-effects model was employed to estimate the pooled effect of taVNS on cognitive outcomes. Between-study variance (*τ*^2^) was estimated using the restricted maximum likelihood (REML) method, which provides an unbiased estimate under conditions of moderate heterogeneity. For verification, results were compared with DerSimonian–Laird (DL) estimators. Within-subject and crossover designs were retained when adequate data were available. When within-subject correlations were not reported, a conservative default correlation of *r* = 0.5 was assumed following established recommendations; sensitivity analyses using *r* values between 0.3 and 0.7 confirmed the stability of the pooled estimates (Lakens, 2013).

Heterogeneity was quantified by the *I*^2^ and *τ*^2^ statistics, and we conducted two sensitivity analyses: (i) Leave-One-Out (LOO) and (ii) Monte-Carlo subset resampling (*k* = 10–30), repeatedly refitting the model to random subsets to assess stability of the pooled effect. To assess small-study and publication bias, Egger’s regression intercept test, the Trim-and-Fill procedure, and the fail-safe *N* Test were performed, with the number of imputed studies (*k*₀) and adjusted pooled estimates reported. We also prespecified a robust analysis of random subsets (repeatedly sampling across different subset sizes to estimate pooled effects) and examined the sources and magnitude of heterogeneity using *τ*^2^–*I*^2^ relationships and effect–*I*^2^ scatter plots.

To enhance inferential robustness, a Bayesian hierarchical random-effects model was additionally implemented using brms. Study-level true effects (*θ* ᵢ) were modeled as normally distributed around a population mean *μ* with between-study variance *τ*^2^. Weakly informative priors were used to regularize estimates while maintaining flexibility: *μ* was assigned a Normal (0, 0.5) prior, reflecting the plausible range of small-to-medium psychological effects, and *τ* followed a Half-Cauchy (0, 0.5) prior. Posterior inference used four chains with 4,000 iterations per chain (2,000 warmup and 2,000 post-warmup), yielding 8,000 posterior draws. Convergence was assessed via *R*^ and adequate bulk/tail effective sample sizes; no convergence issues were indicated. Posterior means and 95% credible intervals (CrI) were summarized for both overall and domain-specific models. All analyses were two-tailed, and statistical significance was defined as *p* < .05 for frequentist estimates or posterior probability *P* (*μ* > 0) > 0.95 for Bayesian models.

### Quality assessment

We evaluated quality assessment for all included studies using the Cochrane Risk of Bias 2 tool (RoB 2) (Sterne et al., 2019). Two reviewers (FL and YL) independently completed the RoB 2 signaling questions after a calibration exercise; disagreements were resolved by discussion. Domain-level and overall judgments followed RoB 2 guidance: low risk of bias, some concerns, or high risk of bias. Overall assessment based on the most unfavorable domain: one high risk indicates overall high; all low domains indicate overall low; all others indicate some concern. The quality assessment results, and visualization are presented in Supplementary Appendix D.

## Results

### Literature search

**Figure 3.**
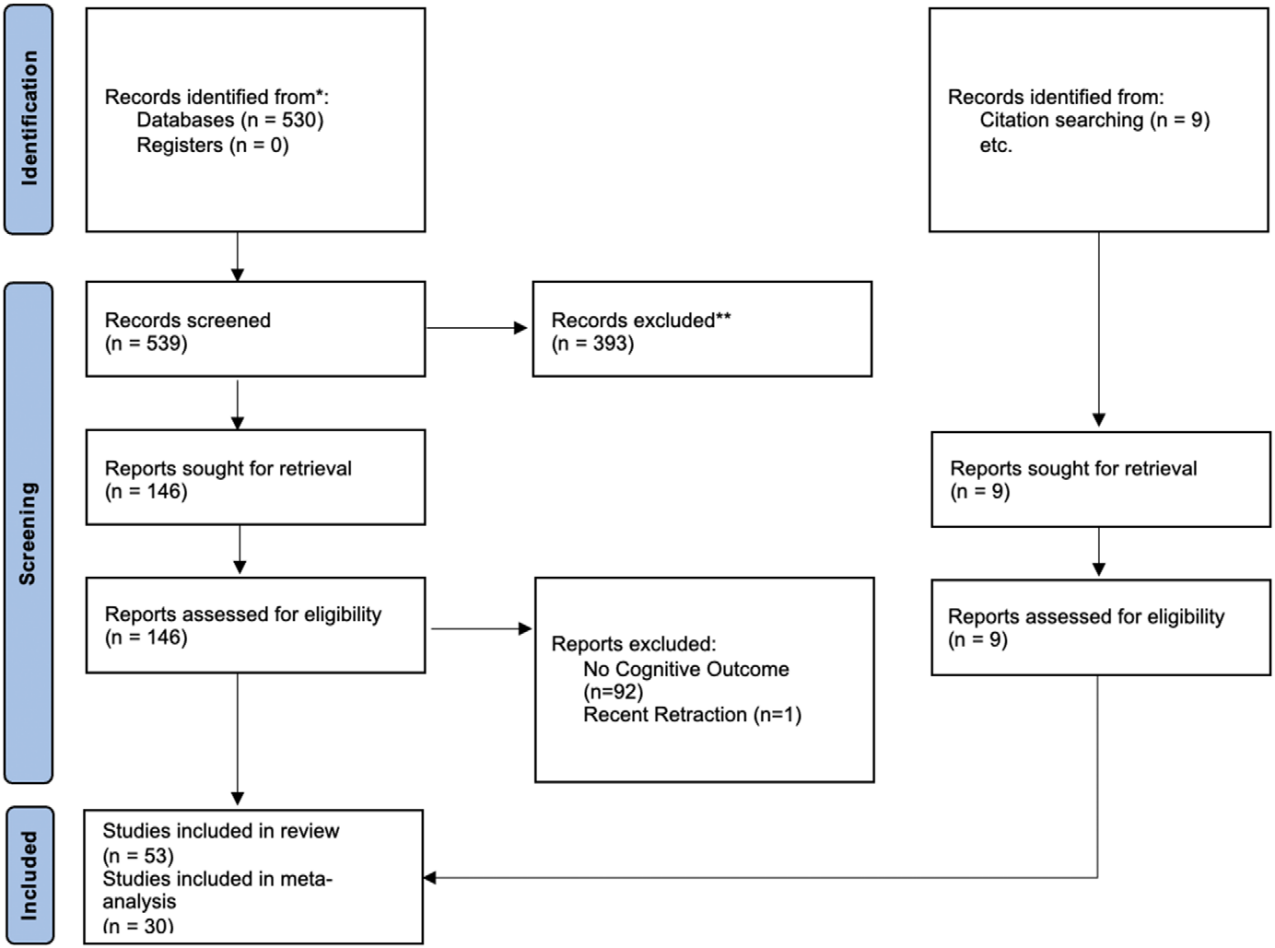
PRISMA flowchart for studies selection and inclusion. Figure 3. long description.

We identified 539 unique records. Of these, 393 were excluded due to content or article type (animal models, *n* = 7; irrelevant topics, *n* = 356; conference abstracts without sufficient outcome data to compute effect sizes, *n* = 6; reviews, *n* = 12; opinions/corrections, *n* = 12). Of the remaining 146 articles, 92 were excluded for not reporting a cognitive outcome, and one was excluded due to a recent retraction. The final sample comprised 53 studies; 30 contributed data to the quantitative meta-analysis (see Figure 3).

### Study characteristics

All studies were published between 2015 and 2025, and sample sizes ranged from 20 to 124 participants (median ≈ 52), encompassing both healthy adults and various clinical populations. Geographically, studies were conducted across Europe (*n* = 29), Asia (*n* = 18), and North/South America (*n* = 6), with most experiments implemented in university or hospital laboratory settings (see Supplementary Appendix E for an overview of study locations and publication-year trends). Regarding stimulation characteristics, taVNS was typically delivered to the left cymba conchae or tragus using auricular electrodes at frequencies between 20 and 30 Hz, intensities of 0.5–1.5 mA, and pulse widths of 200–500 μs. Session durations ranged from 15 to 30 minutes. Earlobe sham stimulation was the most common control condition.

### Qualitative synthesis of the evidence

Across the past decade, taVNS has rapidly evolved from a peripheral neurostimulation technique into a promising cognitive neuromodulation tool. The studies included in this review collectively chart a clear developmental trajectory. Early exploratory trials (2014–2018; e.g. Jacobs et al., 2015; Colzato, Sellaro, & Beste, 2017; Burger et al., 2018) focused on proof-of-principle demonstrations in healthy adults, examining immediate cognitive effects during or shortly after brief stimulation sessions. These studies primarily targeted executive control and conflict monitoring tasks (e.g. Simon, Flanker, and Go/No-Go), consistently reporting improved inhibitory control and post-error adjustment – effects hypothesized to arise from enhanced LC-NE-mediated arousal. Subsequent research (2019–2022) diversified into domain-specific paradigms, investigating working memory, learning, emotion regulation, and social cognition across varied populations (Borges et al., 2020; Tona et al., 2020; Oehrn et al., 2022; Verkuil & Burger, 2019). This second wave began integrating neurophysiological markers (EEG theta, ERP N2/P3, fNIRS oxy-Hb, and pupil dilation) to capture mechanistic indices of vagal engagement. More recently (2023–2025), studies have shifted toward translational and clinical applications in populations such as mild cognitive impairment (Dolphin et al., 2023; Wang et al., 2022) and major depressive disorder (Zhao et al., 2025). Parallel work in healthy participants has begun examining stimulation parameter optimization (e.g. intensity, duty cycle, and electrode site), marking a methodological maturation of the field.

Evidence spanning studies collectively indicates that taVNS is associated with task-dependent, load-sensitive, and state-dependent effects on cognition. While behavioral effects vary in magnitude, neurophysiological alterations (e.g. ERP N2/P3 components, frontal theta, pupil response, HRV, and frontal oxygenation/connectivity) demonstrate greater consistency and frequently precede measurable behavioral improvements. Behavioral facilitation was more readily observed under high-load or resource-constrained conditions, whereas under low-load or ceiling-effect conditions, effects predominantly manifested as optimized ‘neural efficiency’ (e.g. reduced No-Go N2 or shorter N2–P3 interpeak latency), reflecting optimization tendencies in frontoparietal resource allocation and gain control.

Studies targeting conflict/inhibition consistently demonstrate enhanced neural markers of conflict adaptation, post-error deceleration, and inhibitory efficiency. taVNS was associated with enhanced post-conflict adaptation in Simon/Flanker-like tasks and reduced conflict-related N2 amplitude, suggesting upregulation of cognitive control and efficiency gains (Fischer, Ventura-Bort, Hamm, & Weymar, 2018). In Go/No-Go and ‘go/change’ paradigms, taVNS reduces overall errors, diminishes conflict costs, and enhances medial frontal theta (a classic neural marker of executive control) during induced go/stop conflicts (Keute et al., 2020). In the everyday contextualized Executive RT-test, taVNS reduced No-Go N2 without impairing inhibitory performance, signifying ‘equivalent inhibition achieved with reduced neural cost’ (Pihlaja, Failla, Peräkylä, & Hartikainen, 2020). Further research observed significantly reduced inter-peak latencies of the N2–P3 complex in the frontal region during No-Go conditions among a burnout subgroup, alongside reduced errors in the non-burnout group, suggesting accelerated and more efficient executive control processes (Pihlaja & Hartikainen, 2025). It should be noted that a minority of studies, despite lacking behavioral improvements, still exhibited clear neurophysiological changes, supporting a phased effect model of ‘neural changes preceding behavioral changes’ (Fischer, Ventura-Bort, Hamm, & Weymar, 2018).

Research on working memory and attention generally demonstrates load-dependent enhancement and physiological optimization. Following 24-hour sleep deprivation, taVNS significantly improved spatial 3-back accuracy compared to active auricular control, while showing no significant effect on PVT alertness. This indicates selective support for high-load WM rather than general reaction time enhancement (Zhao et al., 2023). In event-related protocols, 3-second pulsed transcutaneous auricular vagus nerve stimulation (taVNS) phase/baseline-dependent modulation of pupillary responses and prolongation of target stimulus reaction times in auditory oddball tasks aligns with the gain control theory of LC–NA, suggesting the importance of ‘event-locked’ neuromodulation timing (Villani et al., 2022). Consistency across such studies incorporating EEG/pupil/HRV metrics supports taVNS’s role in sustaining working memory operations by stabilizing arousal and enhancing signal-to-noise ratios under resource-constrained or declining alertness states.

Regarding emotional and social functioning, taVNS effects vary with task characteristics but consistently demonstrate enhanced prosocial tendencies and improved processing of positive information across multiple models. In a prisoner’s dilemma paradigm among epilepsy patients, taVNS increased cooperation rates within a crossover, double-blind design. Diffusion models revealed early modulation of initial biases, suggesting influence on early decision-making stages and reward/motivation circuits (Oehrn et al., 2022). In contrast, among high-anxiety individuals, taVNS failed to improve inhibitory attention (IOR) toward fearful faces, suggesting a ceiling or mismatch effect in those with high baseline anxiety/potential high LC tension. However, it enhanced IOR toward neutral faces in individuals with high HRV, further emphasizing the modulation of effects by individual baseline states (Verkuil & Burger, 2019). Collectively, these findings reveal taVNS’s regulatory influence on the orbitofrontal-amygdala-insular circuit, supporting its role in facilitating emotional regulation and social responsiveness, particularly within negative or ambiguous emotional contexts.

Regarding flexibility and learning, evidence exhibits task complexity and learning type dependence. Under highly complex task switching and response cascade conditions, taVNS was associated with reduced switching costs and errors (see previous section on executive control outcomes), whereas standard-difficulty task switching more readily yields null results, which is consistent with the load-dependent interpretation of ‘greater difficulty yielding greater efficacy’ (Keute et al., 2020). In associative/language learning, taVNS accelerates novel letter-phoneme relationship acquisition while enhancing automatization and decoding, suggesting its potential to facilitate high-interference encoding and consolidation processes (Thakkar et al., 2020). Reinforcement learning studies present a contrasting picture: in go/no-go reinforcement learning paradigms, taVNS reduced learning rates, particularly following punishment, suggesting it may promote exploration-over-exploitation update strategies. This does not contradict the ‘gain/arousal tuning’ model but reflects strategic trade-offs, supporting its potential mechanism of enhancing prefrontal-amygdala circuit plasticity and long-term memory reappraisal.

### Quantitative synthesis of the evidence

#### RO1: The impact of taVNS and cognitive functions

**Figure 4.**
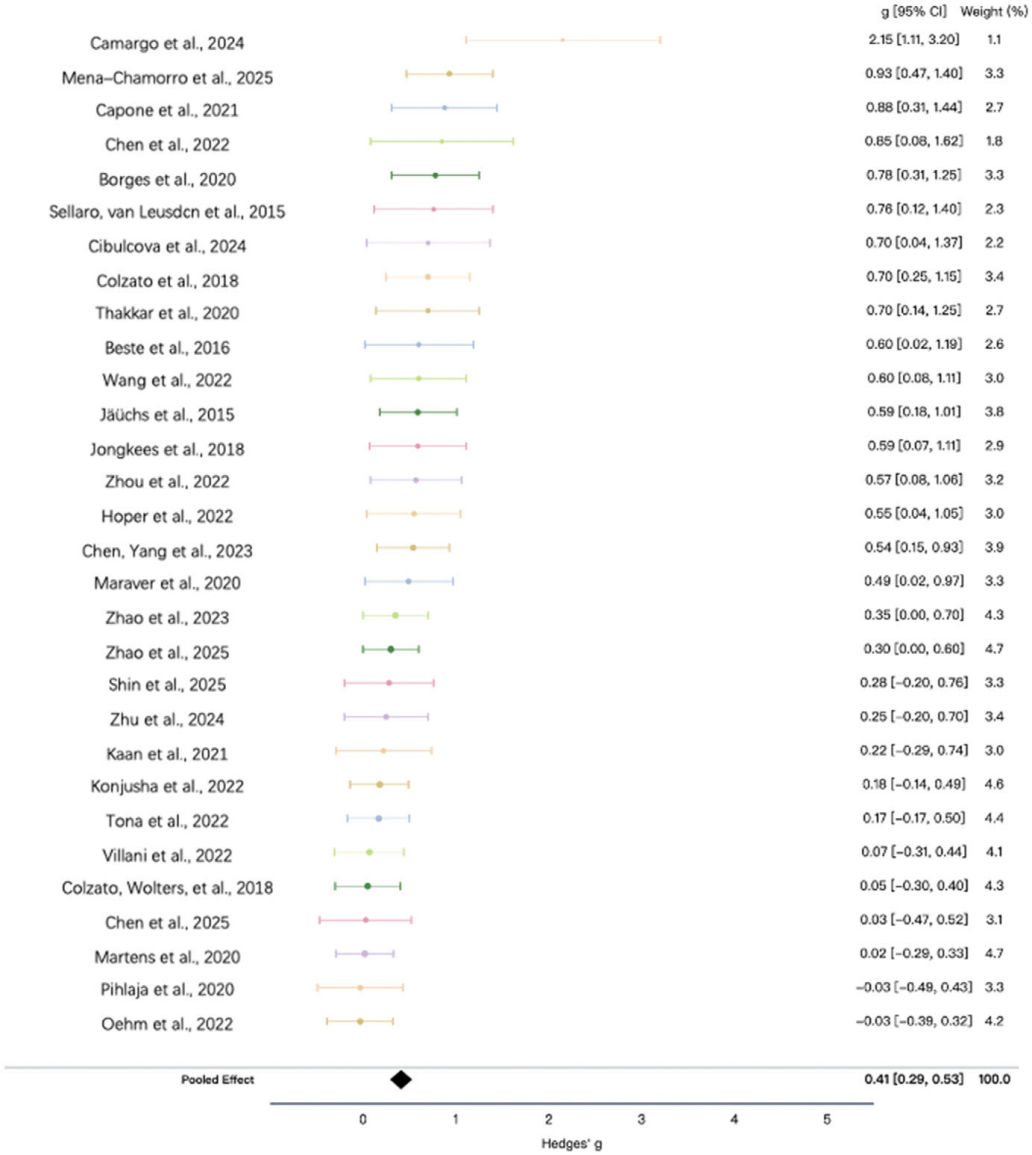
Forest plot of the effects of taVNS on overall cognitive functions. Figure 4. long description.

A random-effects model combining all 30 studies showed a significant, medium-sized positive effect of taVNS on overall cognitive performance (Hedges’ *g* = 0.41, 95% CI: [0.30, 0.53], *p* < .001), with moderate to high heterogeneity (*I*^2^ = 51.4%). The overall effect is visualized in the forest plot (see Figure 4).

### Subgroups in cognitive functions

Subgroup analyses by cognitive function domains indicated broadly consistent benefits of taVNS, with magnitudes varying by construct. For executive functions, a random-effects model across eight studies showed a medium, statistically significant improvement (Hedges’ *g* = 0.46, 95% CI: 0.27–0.65, *p* < .001) and low heterogeneity (*I*^2^ = 9.5%), suggesting fairly concordant effects. Social cognition and emotion regulation yielded a larger point estimate (*k* = 3; *g* = 0.80, 95% CI: 0.07–1.52, *p* = .031) but substantial between-study heterogeneity (*I*^2^ = 82.1%). Working memory and attention showed a small yet reliable advantage (*k* = 9; *g* = 0.19, 95% CI: 0.04–0.33, *p* = .011) with low heterogeneity (*I*^2^ = 14.9%), consistent with a modest but stable effect. Cognitive flexibility and learning demonstrated a medium overall effect (*k* = 10; *g* = 0.53, 95% CI: 0.32–0.75, *p* < .001) with moderate heterogeneity (*I*^2^ = 52.9%), indicating meaningful variability across tasks and protocols. Taken together, taVNS was associated with positive effects across all cognitive functions’ domains (see Table 1 and Supplementary Appendix F, Supplementary Figures F1–F4).

**Table 1.** Overview of pooled effect sizes Table 1. long description.

Summary of pooled effects for taVNS on cognitive functions across four domains					
Cognitive domain	*k*	Hedges’ *g*	95% CI	*I*^2^	*p*-value
Executive functions	8	0.462	[0.272, 0.653]	9.50%	< 0.001
Social cognition and emotional regulation	3	0.795	[0.071, 1.519]	82.10%	0.031
Working memory and attention	9	0.185	[0.043, 0.327]	14.90%	0.011
Cognitive flexibility and learning	10	0.534	[0.324, 0.745]	52.90%	< 0.001

Effect sizes of taVNS across various clinical populations				
Study ID	Clinical condition	Hedges’ *g*	95% CI	Weight (%)
Mena-Chamorro et al., 2025	SCD	0.93	[0.30, 1.57]	11.2
Wang et al., 2022	MCI	0.6	[0.08, 1.11]	10.1
Zhou et al., 2022	CAS	0.57	[0.08, 1.06]	10.4
Dolphin et al., 2023	MCI	0.49	[0.04, 0.94]	12.3
Zhao et al., 2025	MDD	0.3	[−0.01, 0.61]	16.9
Shin et al., 2025	EHL	0.28	[−0.38, 0.94]	10.8
Oehrn et al., 2022	EPI	0.03	[−0.33, 0.39]	14.5

Summary of pooled effect sizes for taVNS across different stimulation intensities					
Intensity subgroup	*k*	Hedges’ *g*	95% CI	*I*^2^	*p*-value
Fixed low intensity (≤1.0 mA)	10	0.207	[−0.038, 0.452]	63.80%	0.098
Fixed high intensity (>1.0 mA)	9	0.575	[0.358, 0.793]	0.00%	< 0.001
Individualized intensity	11	0.461	[0.281, 0.641]	42.60%	< 0.001

### Sensitivity analysis

The LOO analysis demonstrated that the removal of any single study did not significantly alter the overall pooled effect size. The pooled Hedges’ *g* remained stable, with values ranging from 0.39 (when removing Camargo et al., 2024) to 0.43 (when removing Mertens et al., 2020), and the overall effect remained statistically significant in all iterations (all *p* < .001) (see Supplementary Appendix F, Supplementary Figure F5). This confirms that the results are not unduly influenced by any single study.

**Figure 5.**
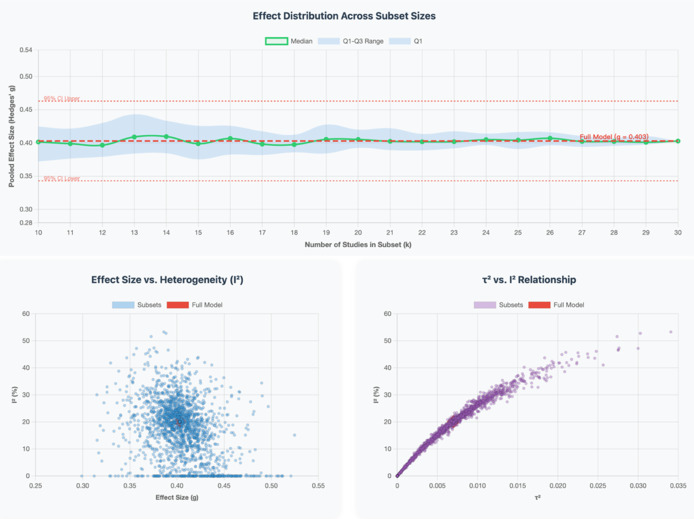
Subsampling sensitivity analysis and heterogeneity diagnostics for the pooled taVNS effect. *Note:* Top: Monte-Carlo subset resampling (*k* = 10–30) shows median pooled Hedges’ *g* tracking the full-model estimate (red dashed line; 95% CI in red dotted lines), with Q1–Q3 ribbon narrowing as *k* increases. Bottom-left: No systematic association between subset effect sizes and heterogeneity (*I*^2^); the full-model point is overlaid in red. Bottom-right: *I*^2^ increases monotonically with *τ*^2^, indicating that observed heterogeneity primarily reflects genuine between-study variance rather than small-study bias. Figure 5. long description.

We also performed a Monte-Carlo subset resampling sensitivity analysis, repeatedly (1,000 runs) fitting the random-effects model to random subsets of size *k* = 10–30; median estimates remained near the full-model value (*g* ≈ 0.40) with narrowing IQRs as *k* increased. All distributions fell within the full model’s 95% CI, indicating the conclusion is not driven by a small number of studies (see the top part of Figure 5).

### Publication bias and small study effects

Visual inspection of the funnel plot revealed some asymmetry. This was statistically confirmed by Egger’s regression test (intercept = 3.91, *p* < .001), suggesting a potential for publication bias favoring studies with positive results. We then applied the trim-and-fill method using the L0 estimator (adjusted *g* = 0.338 [0.220, 0.455]) and conducted Rosenthal’s fail-safe *N* (Nfs = 1067), suggesting the overall conclusion is still robust to a substantial number of unpublished null studies (see Supplementary Appendix F, Supplementary Figure F6 and Supplementary Table F1 for detailed results).

We further examined the relationship between effect size and heterogeneity, along with variance decomposition. Across thousands of random subsets, no systematic correlation was observed between pooled effects and *I*^2^; the scatter plot centered around *g* ≈ 0.40 and *I*^2^ ∼ 10–30%. *I*^2^ exhibited the expected monotonically increasing relationship with *τ*^2^, consistent with *I*^2^ = *τ*^2^/(*τ*^2^ + *v*ˉ); indicating that the observed heterogeneity primarily reflects genuine inter-study variation rather than being attributable to effect inflation or small-sample bias (see in Figure 5 bottom and Supplementary Appendix F, Supplementary Figure F7).

#### RO2: Exploratory impact of taVNS on clinical populations

To specifically investigate the impact of taVNS on individuals with clinical conditions, we conducted a subgroup meta-analysis including the seven studies. The clinical populations encompassed major depressive disorder (MDD), mild cognitive impairment (MCI), and patients at risk for delayed neurocognitive recovery (EPI). Given a small *k* within diagnoses, we aggregated clinical cohorts to stabilize estimation. Subgrouping by individual diagnoses was statistically underpowered. Therefore, all clinical cohorts were analyzed collectively to ensure stable estimation of pooled effects. This choice maximizes precision without over-interpreting sparse strata. A random-effects model showed a significant, medium-to-large positive effect of taVNS on cognitive performance in these clinical populations (Hedges’ *g* = 0.55, 95% CI: [0.31, 0.79], *p* < .001). Heterogeneity within this subgroup was low to moderate (*I^2^* = 29.5%, *τ*^2^ = 0.03).

To formally compare the effect between clinical and healthy populations, a meta-regression was performed with population type as a moderator. The analysis did not show a statistically significant difference in the magnitude of the effect between clinical (*k* = 7) and healthy (*k* = 23) cohorts (*β* = −0.001, *p* = .994). This suggests that the cognitive-enhancing effects of taVNS are comparably effective in both populations (see in Table 1).

#### RO3: Meta-regression analyses of heterogeneity

To explore the sources of inter-study heterogeneity, we conducted three meta-regression analyses on the stimulation intensity, stimulation mode and duration, and stimulation site (see Table 1).

*Stimulation intensity.* A subgroup analysis comparing different intensity strategies showed that studies using fixed high intensity (>1.0 mA) (*g* = 0.58, 95% CI: [0.36, 0.79]) or individualized intensity (*g* = 0.46, 95% CI: [0.28, 0.64]) yielded significant effects, whereas studies using fixed low intensity (≤1.0 mA) did not produce a statistically significant pooled effect (*g* = 0.21, 95% CI: [−0.04, 0.45], *p* = .098).

*Stimulation mode and duration.* Neither the stimulation mode (online vs. offline; *β* = 0.04, *p* = .791) nor the duration of a single stimulation session (*β* = 0.003, *p* = .609) were found to be significant moderators of the effect size.

*Stimulation site.* No significant difference was found between studies stimulating the tragus versus the concha (*β* = 0.03, *p* = .882.).

### REML and DL comparison

To test method robustness, DL estimation was used for re-pooling, yielding highly consistent results with REML: the effect size differences (Δ *g*) across domains were small for both methods (e.g. overall Δ *g* = 0.024; executive function Δ *g* = −0.009; social cognition/emotional regulation Δ *g* = 0.147; working memory/attention Δ *g* = 0.014; cognitive flexibility/learning Δ *g* = 0.032). Correlation between methods reached *r* = .978, 95% CI: [.920, .997], *r^2^* = .957, *p* < .001. Bland–Altman analysis resulted in a mean difference of 0.0416, with 95% consistency limits [−0.0778, 0.1610], indicating minimal systematic bias (see Supplementary Appendix G for detailed results).

### Bayesian meta analyses for enhancing the robustness

Following completion of traditional (frequency-based) random-effects and subgroup/sensitivity analyses, we further employed Bayesian hierarchical modelling for validation due to the following reasons: First, unlike frequentist confidence intervals, Bayesian credible intervals provide direct probabilistic statements about the location of the true effect. Second, the hierarchical structure with weakly informative priors induces partial pooling (shrinkage), which stabilizes effect estimates in domains with small numbers of studies (e.g. social cognition and emotional regulation, *k* = 3) by borrowing strength from the overall distribution of effects; this guards against overconfident conclusions from sparse data while still allowing domain-specific inference. Third, posterior probabilities offer a natural metric for quantifying the evidence that taVNS confers benefit, which is particularly informative under conditions of moderate-to-high heterogeneity where frequentist *p*-values may be difficult to interpret.

### Overall cognitive functions

A Bayesian random-effects meta-analysis across 30 independent comparisons indicated a positive overall effect of taVNS on cognitive functions: *μ* = 0.396, 95% CrI: [0.271, 0.531]. Between-study heterogeneity was moderate: *τ* = 0.253, 95% CrI: [0.122, 0.404]. The posterior probability that the true overall effect is positive was ~1.00, indicating a near-certain benefit on aggregated cognitive outcomes.

### Subgroup analyses by cognitive functions

Prespecified domain models (fit independently) showed heterogeneous patterns across domains. For cognitive flexibility and learning (*k* = 10), evidence indicates a stable, moderate positive effect with low-to-moderate heterogeneity (*μ* = 0.530, 95% CrI: [0.319, 0.757]; *τ* = 0.232, 95% CrI: [0.033, 0.489]; *P* (*μ* > 0) = 0.9999). For executive functions (*k* = 8), results support a moderate, credibly positive effect with low-to-moderate heterogeneity (*μ* = 0.465, 95% CrI: [0.236, 0.701]; *τ* = 0.167, 95% CrI: [0.007, 0.473]; *P* (*μ* > 0) = 0.999). For working memory and attention (*k* = 9). The point estimate suggests a small benefit, though uncertainty includes the null (*μ* = 0.133, 95% CrI: [−0.051, 0.325]; *τ* = 0.162, 95% CrI: [0.007, 0.440]; *P* (*μ* > 0) = 0.931). For social cognition and emotional regulation (*k* = 3), despite a large point estimate, precision is limited (small *k*), and heterogeneity is high. *μ* = 0.626, 95% CrI: [−0.333, 1.521]; *τ* = 0.745, 95% CrI: [0.065, 2.094]; *P* (*μ* > 0) = 0.932.

### Study-level posterior estimates

**Figure 6.**
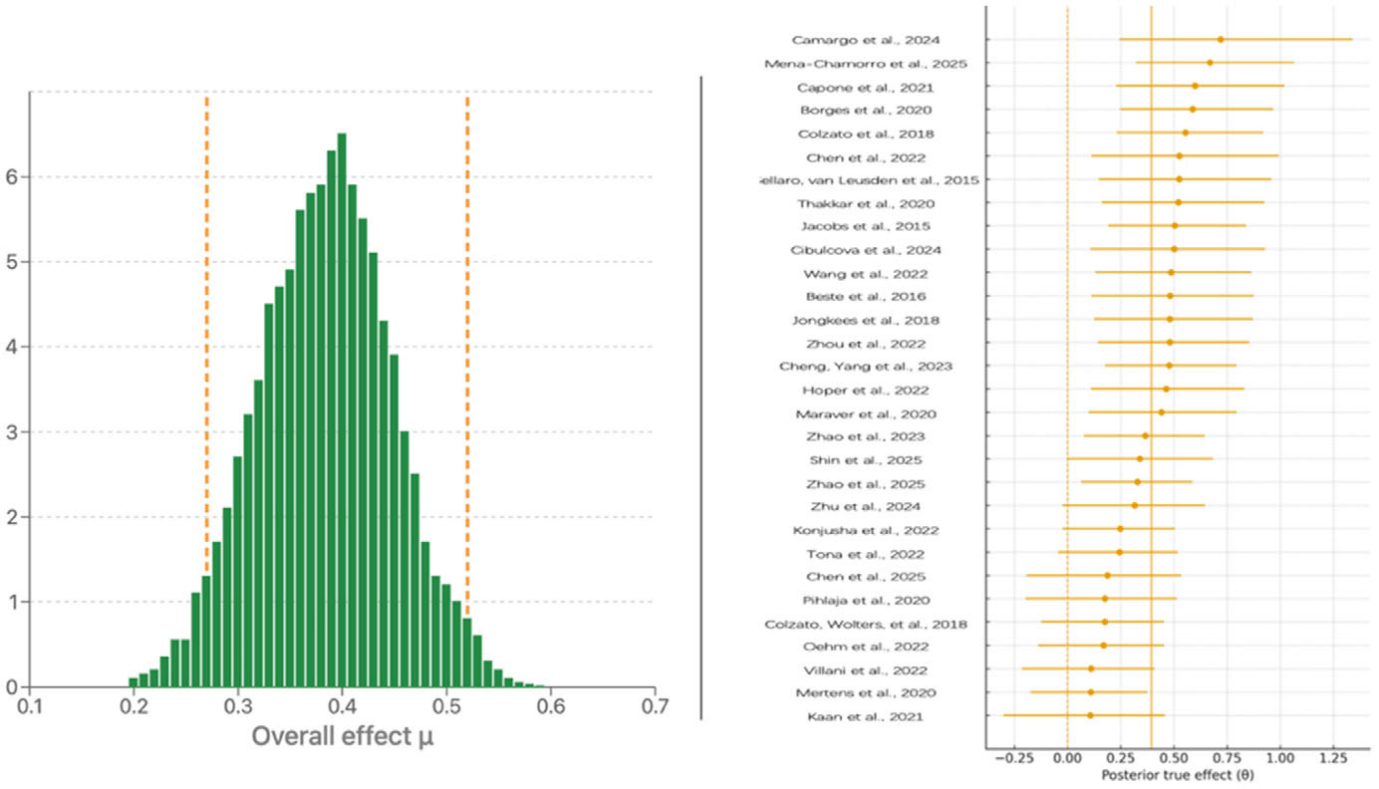
Posterior distribution of the overall effect size (*μ*) and forest plot of posterior means and 95% CrIs for individual studies. Figure 6. long description.

Posterior means and 95% CrIs for each study’s true effect (*θ* ᵢ) are provided in the forest data table (see Supplementary Appendix H, Supplementary Tables H1 and H2) and visualized in the forest plot (see Figure 6). These study-level estimates reflect shrinkage toward the pooled mean proportional to within-study variance and the inferred heterogeneity.

## Discussion

The present systematic review and meta-analysis provide the most comprehensive quantitative synthesis to date of the cognitive effects of taVNS. In sum, taVNS was associated with statistically significant improvement in overall cognitive performance, a finding that remained robust across multiple sensitivity and Bayesian analyses. Domain-specific models further showed a consistent pattern of benefits: moderate, reliable improvements in executive functions and cognitive flexibility and learning, and small but significant gains in working memory and attention. Exploratory analyses also suggested potentially larger effects in social cognition and emotional regulation; however, these findings are based on a limited number of studies (*k* = 3) with substantial heterogeneity and should therefore be interpreted as preliminary pending replication. Importantly, comparable effects were observed in both healthy and clinical populations, suggesting that the potential relevance of taVNS may extend to clinical populations. Together, these results support taVNS as a promising noninvasive neuromodulation technique with the potential to influence multiple cognitive domains through vagal–brainstem–prefrontal circuitry. Extending prior small-scale findings, this synthesis provides a cohesive evidence base suggesting that taVNS may facilitate executive control, adaptive learning, and affective regulation.

To advance a more unified theoretical understanding of how taVNS modulates cognition, we propose the Vagal Neurocognitive Integration Model (VNIM) that links vagal afferent activation with domain-specific cognitive outcomes through shared neuromodulatory mechanisms. This framework conceptualizes taVNS as a bidirectional regulator of brain function, integrating ascending bottom-up vagal inputs with descending top-down cortical control to produce coherent changes across multiple cognitive domains.

At the bottom-up level of VNIM, vagal afferent activation engages the NTS–LC–raphe pathway described above, releasing norepinephrine and serotonin throughout cortical and subcortical networks. Through this mechanism, taVNS is hypothesized to increase cortical signal-to-noise ratio and optimize the brain’s readiness state for information processing, providing the neurophysiological substrate upon which higher cognitive operations depend. Such arousal-based modulation may explain the modest yet consistent enhancements observed in working memory and attention, where stimulation may improve alertness and sustained focus without expanding structural storage capacity.

**Figure 7.**
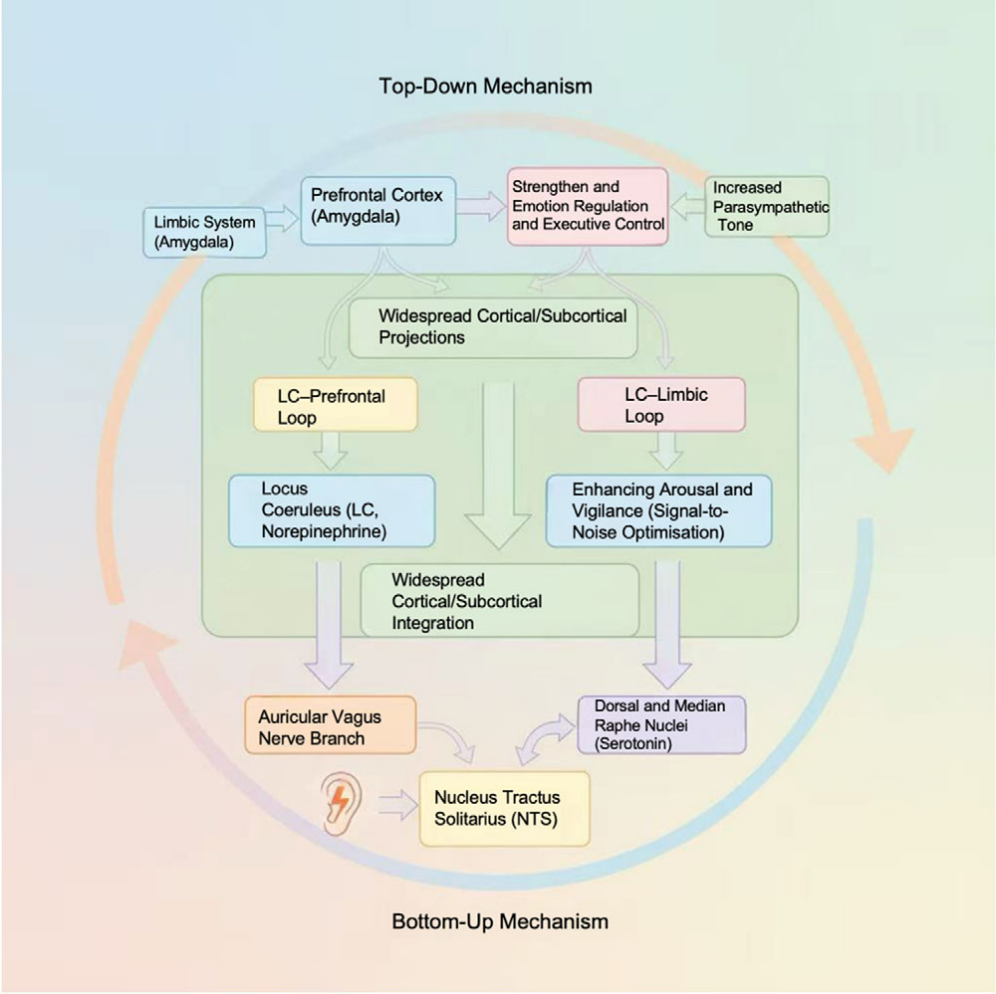
Integrated top-down and bottom-up mechanisms of the vagal neurocognitive integration model. Figure 7. long description.

At the top-down level of VNIM, the LC establishes reciprocal projections to the PFC and limbic regions (Scammell, Arrigoni, & Lipton, 2017). The moderate-to-large effects observed in executive functions and cognitive flexibility are consistent with strengthened LC–NE-driven prefrontal modulation of inhibitory control, conflict monitoring, and adaptive decision-making. Regarding social cognition and emotional regulation, the exploratory findings from a limited number of studies (*k* = 3) suggest potentially larger effects, although the substantial heterogeneity (*I*^2^ = 82.1%) precludes firm conclusions. These preliminary observations are nonetheless consistent with the hypothesized engagement of vagal–limbic circuits: stimulation of the vagal afferents may enhance parasympathetic tone and modulate prefrontal–amygdala coupling, thereby facilitating emotion regulation and empathic processing. The considerable variability in these outcomes likely reflects a combination of methodological differences across studies and individual differences in baseline vagal tone and limbic reactivity, as predicted by the polyvagal framework (Porges, 2009). Critically, these bottom-up and top-down processes are bidirectionally integrated, forming a closed-loop mechanism that continuously regulates arousal–control and reactivity–regulation balance (see Figure 7).

The theoretical interpretation of domain-specific effects further reinforces this integrative view of VNIM. The prominent gains in executive control and flexibility are consistent with the adaptive-gain theory of LC–NE function (Aston-Jones & Cohen, 2005), wherein phasic LC activation amplifies task-relevant neural representations and optimizes behavioral adaptation. By modulating this adaptive gain, taVNS may improve the balance between exploitation and exploration in cognitive control. Conversely, the modest but consistent effects in working memory and attention align with Cowan’s (2001) state-regulation hypothesis: taVNS enhances the neurophysiological state that supports optimal performance rather than altering intrinsic capacity limits. As noted above, the social cognition findings remain exploratory, and individual variability in this domain may be governed by baseline parasympathetic flexibility and vagal–limbic pathway integrity, a hypothesis requiring confirmation from adequately powered studies.

In summary, the VNIM provides a unified framework for interpreting the multi-domain cognitive effects of taVNS. Within the VNIM, taVNS is proposed to act through three primary mechanisms: (i) LC-noradrenergic modulation, which enhances cortical signal-to-noise ratio and stabilizes arousal; (ii) prefrontal control enhancement, supporting inhibition, conflict monitoring, and cognitive flexibility; and (iii) affect regulation via orbitofrontal–amygdala circuitry. These mechanisms may help explain the more consistent effects observed for executive control and cognitive flexibility, while also pointing to three provisional moderators: (i) larger effects under higher task demands; (ii) stronger benefits among individuals with lower baseline arousal/vagal tone; and (iii) dose advantages for above-threshold or individually titrated intensities over fixed low-intensity protocols.

Drawing upon the aggregated evidence and its corresponding certainty ratings, several clinical recommendations can be formulated to guide the translational application of taVNS.

Because taVNS is portable and suitable for home-based administration, it offers potential for scalable translation across two domains: clinical treatment and cognitive optimization in healthy populations. In nonclinical contexts, taVNS may serve as a pre-task intervention to enhance arousal regulation and stabilize performance on cognitively demanding activities. Clinically, VNIM suggested greater responsiveness in conditions marked by control-system inefficiency (e.g. MCI, depression with cognitive slowing, and neurorehabilitation). taVNS may also be considered a promising adjunctive approach for cognitive rehabilitation in MCI, ADHD, and mood disorders presenting with executive dysfunction. Evidence further supports the use of individualized or higher-intensity stimulation protocols (>1 mA) when tolerated, as these parameters have yielded consistently larger effects than fixed low-intensity settings. Conditional recommendations extend to the integration of taVNS within multimodal treatment programs, particularly when combined with pharmacological or cognitive-behavioral therapies in cases where residual cognitive deficits persist.

Patient selection should prioritize individuals with preserved sensory tolerance, intact auricular skin integrity, and adequate motivation for structured follow-up. Stimulation frequencies between 20 and 30 Hz, pulse widths of 200–300 μs, and intensities titrated just below the pain threshold have been most associated with cognitive benefit. Typical treatment sessions last 20–30 minutes, administered three to five times per week. Continuous monitoring of attentional, executive, and affective domains every 4–6 weeks is recommended, complemented by physiological indices such as heart-rate variability to track vagal engagement.

In terms of implementation, taVNS can be delivered across a variety of settings, ranging from outpatient clinics to home-based, tele-supervised environments. Incorporating taVNS into care pathways should be pragmatic and evidence-proportionate. Clinicians may consider positioning taVNS within stepped-care algorithms for cognitive dysfunction after reversible causes are optimized. Finally, equity and access warrant attention: as consumer devices proliferate, evidence-based guidance is needed to avoid widening socioeconomic disparities in access to neuromodulatory care.

Despite the robustness of the present findings, several limitations warrant consideration. First, because relatively small clinical samples were available, conducting separate analyses for each diagnostic category would have resulted in insufficient statistical power. Accordingly, we aggregated all clinical cohorts into a unified subgroup to obtain a more stable and interpretable pooled estimate. This approach is methodologically justified, given the consistent outcomes across sensitivity analyses. However, future investigations should aim to enlarge the clinical evidence base through adequately powered, diagnosis-specific trials to clarify potential differences in taVNS responsiveness across clinical populations. Second, some key mechanistic pathways remain incompletely understood. While current neuroimaging and electrophysiological data implicate the locus coeruleus–prefrontal network, the precise causal chain linking vagal activation to specific cognitive domains remains inferential, and the moderating roles of baseline cognitive reserve and affective state require further examination. Third, the interpretation of taVNS effects must consider the methodological challenge of incomplete blinding inherent to auricular stimulation paradigms. Although most included studies employed earlobe sham stimulation, perceptible sensory differences between active and sham conditions (e.g. tingling, warmth, or pulsatile sensations at the cymba conchae or tragus versus the earlobe) may compromise participant blinding and introduce expectancy-related effects. Although several studies reported successful blinding based on post-session allocation guessing, this approach may underestimate partial unblinding or subtle expectancy influences. Future trials should systematically assess and report blinding integrity using validated instruments, incorporate active sham conditions that more closely match the sensory profile of verum stimulation (e.g. low-intensity stimulation at non-vagal auricular sites), and consider statistical adjustment for expectancy covariates where feasible.

Building upon these limitations, future research should prioritize several directions. First, mechanistic convergence should be sought through multimodal imaging techniques – such as simultaneous EEG–fMRI coupling, pupillometry, and neurometabolic tracing – to delineate the locus coeruleus–norepinephrine and cholinergic signatures that may underlie the observed cognitive effects. Second, dose–response optimization using adaptive, closed-loop stimulation paradigms is warranted to dynamically adjust parameters in real time according to individual neurophysiological feedback. Third, longitudinal and ecologically valid trials are needed to evaluate the durability, generalizability, and behavioral transfer of taVNS-induced improvements to everyday cognitive functioning. Finally, comparative efficacy studies against established interventions, including tDCS, TMS, and pharmacological cognitive enhancers, should determine relative benefit, scalability, and cost-effectiveness. Together, these avenues may help advance taVNS from an emerging research technique toward a more clinically mature, mechanism-informed approach to cognitive support and neuropsychiatric rehabilitation.

## Supporting information

10.1017/S0033291726105017.sm001 Liu and Li supplementary material Liu and Li supplementary material

## Supplementary material

The supplementary material for this article can be found at http://doi.org/10.1017/S0033291726105017.

## Long descriptions

### Figure 1. Long description

The left panel shows a profile of a human head with a stimulation arrow pointing to a yellow circle at the ear. Lines trace from this point to the N T S in the brainstem. From the N T S, neural pathways radiate upward into the brain, labeled with Prefrontal Cortex P F C for executive control, norepinephrine N E release, serotonin 5 H T release, and the limbic system for emotion and memory.

The right panel is a flowchart. At the center is a box labeled N T S and S p V forward slash S p 5.

Upward afferent pathways from the N T S include:

* A solid line to D L P F C forward slash P F C.

* A dashed line to D R N forward slash M R N 5 H T.

* A dotted line to P B N, which then connects to the Hippocampus forward slash Amygdala.

* A dashed line also connects the N T S to the A C C forward slash Insula.

* An arrow points from the N T S back toward the A B V N sensory and cervical vagus efferent via plus or minus S p 5.

Downward efferent pathways from the N T S include:

* A dash-dot line labeled A C h efferents forward slash anti-inflammatory pathway leading to a box labeled D M V forward slash N A vagal efferents, A C H.

* This box points further down to a box labeled Viscera.

A legend in the bottom right defines the line styles: solid for N E L C relay, dashed for 5 H T D R N forward slash M R N relay, dotted for P B N relay, and dash-dot for A C H efferents.

Navigate back to Figure 1..

### Figure 2. Long description

At the center is a cross-section of a human brain with labeled regions including the Amygdala, Insula, P F C, A C C, and Parietal Lobe. A vertical blue line representing the Auricular Vagus Nerve Branch ascends through the brainstem, branching out as Vagal Afferent Pathways to these regions.

Surrounding the brain are four castle icons representing cognitive domains:

* Top-Left: A red castle with a lion crest labeled Executive Functions, including Decision-Making, Planning, and Impulse Control. It is linked to the P F C.

* Top-Right: A blue castle with an eagle crest labeled Working Memory and Attention, linked to the prefrontal-parietal network.

* Bottom-Right: A green castle with a snake crest labeled Cognitive Flexibility and Learning, linked to Locus Coeruleus-Norepinephrine L C N E Modulation.

* Bottom-Left: A yellow castle with a badger crest labeled Social Cognition and Emotional Regulation, linked to the Amygdala P F C Insula Network.

Arrows indicate Modulation via Brainstem Vagal Pathways connecting the central nerve branch to these external domains.

Navigate back to Figure 2..

### Figure 3. Long description

The flowchart is organized into three vertical phases labeled on the far left.

1. Identification Phase.

Top left box: Records identified from Databases n equals 530 and Registers n equals 0.

Top right box: Records identified from Citation searching n equals 9 etc.

2. Screening Phase.

Left column sequence:

- Records screened n equals 539. An arrow points right to a box for Records excluded n equals 393.

- Reports sought for retrieval n equals 146.

- Reports assessed for eligibility n equals 146. An arrow points right to a box for Reports excluded: No Cognitive Outcome n equals 92 and Recent Retraction n equals 1.

Right column sequence:

- Reports sought for retrieval n equals 9.

- Reports assessed for eligibility n equals 9.

3. Included Phase.

Bottom box: Arrows from both the left and right screening columns converge here. Studies included in review n equals 53. Studies included in meta-analysis n equals 30.

Navigate back to Figure 3..

### Figure 4. Long description

The forest plot lists 30 studies on the y-axis and Hedges’ g values on the x-axis ranging from negative 1 to 5.

Top entries include Camargo et al. 2024 with the largest effect of 2.15 and a 95 percent C I of 1.11 to 3.20. Subsequent studies like Mena-Chamorro et al. 2025 and Capone et al. 2021 show decreasing effect sizes of 0.93 and 0.88 respectively.

The middle section contains studies such as Wang et al. 2022 with 0.60 and Zhao et al. 2023 with 0.35. As the list descends, the effect sizes approach zero, with Pihlaja et al. 2020 and Oehm et al. 2022 both showing a slight negative effect of negative 0.03.

Each study is represented by a colored dot for the point estimate and horizontal whiskers for the confidence interval. At the bottom, a black diamond represents the Pooled Effect. The center of the diamond aligns with a Hedges’ g of 0.41, with the width representing a 95 percent C I of 0.29 to 0.53. This pooled result is statistically significant as the diamond does not cross the vertical line of null effect at zero. The total weight is 100.0 percent.

Navigate back to Figure 4..

### Table 1. Long description

The figure consists of three forest plots analyzing the effects of t a V N S. All plots use Hedges’ g as the effect size measure on the x-axis, ranging from negative 1.0 to 2.0.

Top Panel: Summary of pooled effects across four cognitive domains.

1. Executive functions: k equals 8, Hedges’ g is 0.462, 95% C I [0.272, 0.653], I-squared is 9.50%, p-value less than 0.001.

2. Social cognition and emotional regulation: k equals 3, Hedges’ g is 0.795, 95% C I [0.071, 1.519], I-squared is 82.10%, p-value is 0.031.

3. Working memory and attention: k equals 9, Hedges’ g is 0.185, 95% C I [0.043, 0.327], I-squared is 14.90%, p-value is 0.011.

4. Cognitive flexibility and learning: k equals 10, Hedges’ g is 0.534, 95% C I [0.324, 0.745], I-squared is 52.90%, p-value less than 0.001.

Middle Panel: Effect sizes across clinical populations.

1. Mena-Chamorro et al. (S C D): g is 0.93, 95% C I [0.30, 1.57], weight 11.2%.

2. Wang et al. (M C I): g is 0.6, 95% C I [0.08, 1.11], weight 10.1%.

3. Zhou et al. (C A S): g is 0.57, 95% C I [0.08, 1.06], weight 10.4%.

4. Dolphin et al. (M C I): g is 0.49, 95% C I [0.04, 0.94], weight 12.3%.

5. Zhao et al. (M D D): g is 0.3, 95% C I [-0.01, 0.61], weight 16.9%.

6. Shin et al. (E H L): g is 0.28, 95% C I [-0.38, 0.94], weight 10.8%.

7. Oehrn et al. (E P I): g is 0.03, 95% C I [-0.33, 0.39], weight 14.5%.

Bottom Panel: Summary of pooled effect sizes across stimulation intensities.

1. Fixed low intensity (less than or equal to 1.0 m A): k equals 10, g is 0.207, 95% C I [-0.038, 0.452], I-squared is 63.80%, p-value is 0.098.

2. Fixed high intensity (greater than 1.0 m A): k equals 9, g is 0.575, 95% C I [0.358, 0.793], I-squared is 0.00%, p-value less than 0.001.

3. Individualized intensity: k equals 11, g is 0.461, 95% C I [0.281, 0.641], I-squared is 42.60%, p-value less than 0.001.

Navigate back to Table 1..

### Figure 5. Long description

The top panel is a line graph titled Effect Distribution Across Subset Sizes. The x-axis is Number of Studies in Subset k from 10 to 30. The y-axis is Pooled Effect Size Hedges g from 0.28 to 0.54. A green line with dots represents the median, which fluctuates around 0.40. A red dashed line marks the Full Model g equals 0.403. Red dotted lines show the 95 percent C I Upper and Lower bounds. A light blue ribbon representing the Q 1 to Q 3 range narrows as k increases.

The bottom-left panel is a scatter plot titled Effect Size versus Heterogeneity I super 2. The x-axis is Effect Size g from 0.25 to 0.55. The y-axis is I super 2 percent from 0 to 60. Blue dots represent subsets clustered around the center. A single red dot marks the Full Model at approximately g equals 0.40 and I super 2 equals 20 percent.

The bottom-right panel is a scatter plot titled tau squared versus I super 2 Relationship. The x-axis is tau squared from 0 to 0.035. The y-axis is I super 2 percent from 0 to 60. Purple dots show a monotonic logarithmic-style increase where I super 2 rises sharply at low tau squared values and then begins to plateau. A red dot represents the Full Model positioned along this curve.

Navigate back to Figure 5..

### Figure 6. Long description

The left panel is a histogram titled Overall effect mu on the x-axis, which ranges from 0.1 to 0.7. The y-axis represents density from 0 to 6. A green bell-shaped distribution peaks at approximately 0.4. Two vertical dashed orange lines mark the 95 percent credible interval boundaries at roughly 0.27 and 0.52.

The right panel is a forest plot with the x-axis labeled Posterior true effect theta ranging from negative 0.25 to 1.25. A vertical solid orange line represents the mean overall effect at 0.4, and a vertical dashed orange line marks the zero point. Thirty-one individual studies are listed vertically on the y-axis, starting from Camargo et al., 2024 at the top to Kaan et al., 2021 at the bottom. Each study is represented by an orange dot for the posterior mean and a horizontal orange line for the 95 percent C r I. Most studies show a positive effect, with means clustered between 0.1 and 0.7, though several lower studies have intervals that cross the zero line.

Navigate back to Figure 6..

### Figure 7. Long description

The diagram is organized into three main horizontal sections connected by a large circular arrow indicating a continuous cycle.

At the top, the Top-Down Mechanism section contains four boxes in a linear sequence from left to right. Limbic System Amygdala points to Prefrontal Cortex Amygdala, which points to Strengthen and Emotion Regulation and Executive Control, which finally points to Increased Parasympathetic Tone.

The central green box represents the integration zone. At its top is Widespread Cortical slash Subcortical Projections. Below this, two parallel vertical paths exist. On the left, L C dash Prefrontal Loop points down to Locus Coeruleus L C, Norepinephrine. On the right, L C dash Limbic Loop points down to Enhancing Arousal and Vigilance Signal dash to dash Noise Optimisation. Both paths converge at the bottom of the green box into Widespread Cortical slash Subcortical Integration.

At the bottom, the Bottom-Up Mechanism section shows an icon of an ear with a lightning bolt pointing to Auricular Vagus Nerve Branch. This points to Nucleus Tractus Solitarius N T S. To the right, Dorsal and Median Raphe Nuclei Serotonin also points toward the N T S.

Large curved arrows on the perimeter connect these sections. A blue and orange arrow curves downward from the top-right Increased Parasympathetic Tone toward the bottom-up section. A pink and orange arrow curves upward from the bottom-left Auricular Vagus Nerve Branch back toward the top-down section.

Navigate back to Figure 7..
