# Supplementary material for: Sorting the mind: cognitive enhancement through transcutaneous auricular vagus nerve stimulation: a systematic review and meta-analysis
Source: Psychol Med. 2026 Jun 24;56:e207. doi: 10.1017/S0033291726105017 (PMC13319486; doi:10.1017/S0033291726105017)
Supplement: Liu and Li supplementary material [file S0033291726105017sup001.zip › PM_Appendix B_Table B1-4.docx]

**Appendix B**

**Table B1.**

**Search Terms Overview**

| **Category** | **Keywords** |
| --- | --- |
| **Primary Terms** | “transcutaneous auricular vagus nerve stimulation”; “taVNS”; “auricular vagus nerve stimulation”; “transcutaneous vagus nerve stimulation”; “external vagus nerve stimulation”; “vagus nerve stimulation auricular” |
| **Executive Functions** | “executive function*”; “decision-making”; “impulse control”; “planning”; “problem-solving”; “self-regulation”; “inhibition”; “cognitive flexibility”; “cognitive control”; “working memory” |
| **Working Memory & Attention** | “working memory”; “short-term memory”; “attention”; “sustained attention”; “focused attention”; “cognitive attention”; “selective attention”; “divided attention”; “n-back task”; “Stroop test”; “digit span” |
| **Social Cognition & Emotion Regulation** | “social cognition”; “emotion regulation”; “emotion recognition”; “social interaction”; “affective processing”; “emotional intelligence”; “affective regulation”; “emotional control”; “social decision-making” |
| **Cognitive Flexibility & Learning** | “cognitive flexibility”; “learning ability”; “adaptability”; “task-switching”; “set-shifting”; “information processing”; “learning strategies”; “problem-solving adaptability” |
| **Clinical Populations — Cognitive Impairment & Disorders** | “cognitive impairment”; “Alzheimer’s disease”; “ADHD”; “depression”; “neurodegenerative disorder*”; “dementia”; “memory disorders”; “neuropsychological disorders”; “mild cognitive impairment (MCI)”; “executive dysfunction” |
| **Clinical Populations — Other Conditions** | “epilepsy”; “chronic pain”; “autism spectrum disorder”; “schizophrenia”; “post-stroke rehabilitation”; “tinnitus”; “stroke rehabilitation”; “traumatic brain injury (TBI)” |
| **Study Types** | “randomized controlled trial”; “RCT”; “clinical trial”; “observational study”; “cross-sectional study”; “longitudinal study”; “pre-post intervention study”; “controlled trial”; “pilot study”; “case-control study” |
| **Meta-analysis & Systematic Review** | “meta-analysis”; “systematic review”; “pooled analysis”; “effect size”; “quantitative review”; “evidence synthesis” |
| **taVNS Parameters** | “vagus nerve stimulation frequency”; “taVNS intensity”; “taVNS duration”; “vagus nerve stimulation pulse width”; “taVNS session length”; “vagus nerve stimulation protocol”; “taVNS stimulation parameters”; “vagus nerve stimulation safety”; “taVNS side effects” |
| **General Cognitive/Neuromodulation** | “neuromodulation”; “neuroplasticity”; “cognition”; “brain stimulation”; “brain–cognition relationship”; “non-invasive stimulation”; “neurostimulation”; “neurofeedback”; “electrical stimulation” |

Note. The asterisk (*) denotes a truncation/wildcard (e.g., function retrieves functions, functioning). Abbreviations: taVNS = transcutaneous auricular vagus nerve stimulation; RCT = randomized controlled trial; MCI = mild cognitive impairment; TBI = traumatic brain injury; ADHD = attention-deficit/hyperactivity disorder.

**Table B2.**

**Example Boolean Search Strings**

| **#** | **Boolean Combination (for PubMed/PsycINFO/Scopus)** |
| --- | --- |
| 1 | (“transcutaneous auricular vagus nerve stimulation” OR “taVNS” OR “auricular vagus nerve stimulation”) AND (“executive function*” OR “decision-making” OR “problem-solving” OR “impulse control”) |
| 2 | (“taVNS” OR “vagus nerve stimulation”) AND (“working memory” OR “attention” OR “n-back task” OR “Stroop test”) |
| 3 | (“transcutaneous vagus nerve stimulation” OR “taVNS”) AND (“social cognition” OR “emotion regulation” OR “emotion recognition”) |
| 4 | (“taVNS” OR “vagus nerve stimulation”) AND (“cognitive flexibility” OR “learning ability” OR “task-switching”) |
| 5 | (“taVNS” OR “vagus nerve stimulation”) AND (“depression” OR “ADHD” OR “cognitive impairment” OR “Alzheimer’s disease”) |

**Table B3.**

**Inclusion and Exclusion Criteria for Study Selection**

| **Category** | **Inclusion Criteria** | **Exclusion Criteria** |
| --- | --- | --- |
| **Study Type** | • Randomised controlled trials (RCTs), quasi-experimental studies, observational studies, and clinical trials.  • Pre- and post-intervention designs (both longitudinal and cross-sectional).  • Studies including a control group (active or sham) or reporting pre–post intervention comparisons. | • Case reports, reviews, commentaries, and editorials.  • Studies without a control or baseline comparison (e.g., single-group pre-intervention only). |
| **Population** | • No age or ethnic restriction. | • Animal or non-human studies. |
| **Intervention** | • Studies investigating taVNS as the main intervention, regardless of stimulation parameters (e.g., intensity, frequency, duration, intervention period). | • Studies evaluating non-taVNS interventions (e.g., pharmacological treatments, other neuromodulation methods) without sufficient taVNS-specific information. |
| **Outcome Measures** | • Validated measures of cognitive function (e.g., self-report scales, neuropsychological tests, or behavioural tasks).  • Clinical measures of cognitive impairment when includes clinical population (e.g., *Alzheimer’s Disease Assessment Scale – Cognitive Subscale*, *Wechsler Adult Intelligence Scale*). | • Studies not using objective cognitive tests or not clearly reporting relevant cognitive outcomes. |
| **Language** | • No language restriction (translations conducted where possible). | — |
| **Publication Type** | • Peer-reviewed journal articles, dissertations, conference abstracts (if sufficient methodological details provided), and registered clinical trials. | — |
| **Other Exclusions** | — | • Studies lacking clear description of taVNS stimulation parameters (e.g., frequency, intensity, or duration). |

**Table B4.
Study Characteristics and Data Extraction Framework**

| **Category** | **Description** |
| --- | --- |
| **Study Characteristics** | Study ID: Author(s) and publication year.  Study Design: Type of study.  Sample Size: Total sample and subgroup sizes for intervention and control groups.  Duration of Study: Total study length and follow-up periods (if applicable).  Country and Setting: Geographic location and study context. |
| **Participant Characteristics** | Demographics: Age, gender distribution, and other relevant variables.  Clinical Population: Diagnostic category or condition (e.g., ADHD, depression, Alzheimer’s disease). |
| **Intervention Characteristics** | taVNS Parameters: Frequency (Hz), intensity (mA), duration per session (minutes), total intervention period.  Mode of Stimulation: Auricular or transcutaneous application.  Control Group/Comparison: Type of control condition (active, sham, alternative intervention, or wait-list control). |
| **Outcome Measures** | Primary Cognitive Domains: executive function, attention, memory, social cognition, emotional regulation, and cognitive flexibility.  Specific Tests Used: e.g., *Stroop Task*, *Wisconsin Card Sorting Test*, *Digit Span*, *Emotional Regulation Scale*.  Secondary Outcomes: Additional outcomes such as mood, motivation, or quality of life.  Time Points of Measurement: Pre- and post-intervention, and follow-up assessments (if applicable). |
| **Statistical Information** | Reported effect sizes (Cohen’s *d*, Hedges’ *g*, etc.) with corresponding 95% confidence intervals.  Reported significance tests (e.g., *p* values, *t* or *F* statistics). |
