# Supplementary material for: Sorting the mind: cognitive enhancement through transcutaneous auricular vagus nerve stimulation: a systematic review and meta-analysis
Source: Psychol Med. 2026 Jun 24;56:e207. doi: 10.1017/S0033291726105017 (PMC13319486; doi:10.1017/S0033291726105017)
Supplement: Liu and Li supplementary material [file S0033291726105017sup001.zip › PM_Appendix D_Risk of Bias Results .docx]

**Table D1.**

**Risk of Bias Results of 53 Included Studies**

| **Study** | **D1** | **D2** | **D3** | **D4** | **D5** | **Overall** |
| --- | --- | --- | --- | --- | --- | --- |
| Beste et al. 2016 | Low Risk | Low Risk | Some Concerns | Low Risk | Low Risk | High Risk |
| Borges et al. 2020 | Some Concerns | High Risk | Low Risk | Low Risk | High Risk | Low Risk |
| Bretheron et al. 2019 | Low Risk | Low Risk | Some Concerns | Low Risk | Some Concerns | Low Risk |
| Burger et al. 2018 | Low Risk | High Risk | High Risk | Some Concerns | Low Risk | Low Risk |
| Burger et al. 2019 | Low Risk | Some Concerns | Low Risk | Low Risk | Some Concerns | Low Risk |
| Camargo et al. 2024 | High Risk | Some Concerns | Some Concerns | Low Risk | High Risk | Low Risk |
| Capone et al. 2021 | Low Risk | Low Risk | Low Risk | Low Risk | Low Risk | Low Risk |
| Chen et al. 2022 | Low Risk | Some Concerns | Low Risk | High Risk | Some Concerns | Low Risk |
| Chen et al. 2023 | Low Risk | Some Concerns | High Risk | Low Risk | Some Concerns | Some Concerns |
| Chen et al. 2024 | Low Risk | Low Risk | Some Concerns | Low Risk | Low Risk | Some Concerns |
| Chen et al. 2025 | Some Concerns | Low Risk | Some Concerns | Low Risk | Some Concerns | Low Risk |
| Chen, Yang et al. 2023 | Some Concerns | Some Concerns | Some Concerns | Some Concerns | Some Concerns | Low Risk |
| Cibulcova et al. 2024 | Low Risk | Low Risk | Low Risk | Some Concerns | Low Risk | Low Risk |
| Colzato, Wolters, et al. 2018 | Some Concerns | High Risk | Low Risk | Low Risk | Low Risk | Some Concerns |
| Colzato et al. 2017 | Low Risk | Some Concerns | High Risk | High Risk | Some Concerns | Some Concerns |
| Colzato et al. 2018 | Low Risk | Some Concerns | Low Risk | High Risk | Low Risk | Low Risk |
| D'Agostini et al. 2025 | Low Risk | High Risk | Low Risk | Low Risk | High Risk | Low Risk |
| Dastoor et al. 2025 | Some Concerns | High Risk | Some Concerns | High Risk | Some Concerns | Low Risk |
| De Smet et al. 2021 | Low Risk | Some Concerns | High Risk | High Risk | Some Concerns | Low Risk |
| Dolphin et al. 2023 | Some Concerns | Low Risk | Low Risk | Low Risk | Some Concerns | Low Risk |
| Drost et al. 2025 | Some Concerns | Low Risk | Low Risk | Some Concerns | Some Concerns | Low Risk |
| Finisguerra et al. 2019 | Low Risk | Some Concerns | Some Concerns | Low Risk | Low Risk | Low Risk |
| Fischer et al. 2018 | Low Risk | Some Concerns | Some Concerns | Low Risk | Low Risk | Some Concerns |
| Francesco et al. 2025 | Low Risk | High Risk | Some Concerns | Low Risk | Low Risk | High Risk |
| Hoper et al. 2022 | Some Concerns | Some Concerns | High Risk | Low Risk | High Risk | High Risk |
| Jacobs et al. 2015 | High Risk | Low Risk | High Risk | Low Risk | High Risk | Some Concerns |
| Jongkees et al. 2018 | Low Risk | Some Concerns | High Risk | Some Concerns | Some Concerns | Low Risk |
| Kaan et al. 2021 | Low Risk | Low Risk | High Risk | High Risk | Some Concerns | Some Concerns |
| Keute et al. 2020 | Low Risk | High Risk | Low Risk | High Risk | Low Risk | Some Concerns |
| Kongjusha et al. 2022 | High Risk | High Risk | High Risk | Low Risk | Low Risk | Some Concerns |
| Kuhnel et al. 2020 | Some Concerns | Some Concerns | Some Concerns | Low Risk | Low Risk | Some Concerns |
| Maraver et al. 2020 | Some Concerns | High Risk | Some Concerns | Some Concerns | Low Risk | High Risk |
| Mena-Chamorro et al. 2025 | Low Risk | High Risk | High Risk | Low Risk | Low Risk | Some Concerns |
| Mertens et al. 2020 | Low Risk | High Risk | High Risk | Low Risk | High Risk | Some Concerns |
| Oehrn et al. 2022 | Low Risk | High Risk | Low Risk | Low Risk | Low Risk | High Risk |
| Pan et al. 2024 | Low Risk | High Risk | Low Risk | Low Risk | Low Risk | Low Risk |
| Pihlaja et al. 2020 | Low Risk | Low Risk | Some Concerns | Low Risk | Some Concerns | Some Concerns |
| Pihlaja et al. 2025 | Low Risk | Low Risk | Low Risk | High Risk | High Risk | Some Concerns |
| Sellaro, Steenbergen et al. 2015 | Low Risk | High Risk | Low Risk | Low Risk | Low Risk | Low Risk |
| Sellaro, van Leusden et al. 2015 | Low Risk | Low Risk | Low Risk | Low Risk | Low Risk | High Risk |
| Shin et al. 2025 | Some Concerns | High Risk | Some Concerns | Some Concerns | Low Risk | Low Risk |
| Szeska et al. 2020 | Low Risk | Some Concerns | Low Risk | Low Risk | Some Concerns | Low Risk |
| Thakkar et al. 2020 | Low Risk | Some Concerns | Some Concerns | Low Risk | Low Risk | Some Concerns |
| Tona et al. 2022 | Some Concerns | Some Concerns | High Risk | Some Concerns | High Risk | High Risk |
| Verkuil & Burger, 2019 | Low Risk | Some Concerns | High Risk | Low Risk | Some Concerns | Low Risk |
| Villani et al. 2022 | Low Risk | Some Concerns | Some Concerns | Low Risk | High Risk | High Risk |
| Wang et al. 2022 | Some Concerns | Low Risk | Some Concerns | Some Concerns | High Risk | Some Concerns |
| Warren et al. 2020 | Some Concerns | High Risk | Low Risk | Low Risk | Some Concerns | Low Risk |
| Yildiz et al. 2022 | Low Risk | High Risk | Low Risk | High Risk | Low Risk | Low Risk |
| Zhao et al. 2023 | Some Concerns | High Risk | Some Concerns | Low Risk | Low Risk | Low Risk |
| Zhao et al. 2025 | Some Concerns | Low Risk | Some Concerns | Low Risk | High Risk | Low Risk |
| Zhou et al. 2022 | Low Risk | Low Risk | Low Risk | High Risk | Some Concerns | Low Risk |
| Zhu et al. 2024 | Some Concerns | High Risk | High Risk | High Risk | High Risk | Some Concerns |


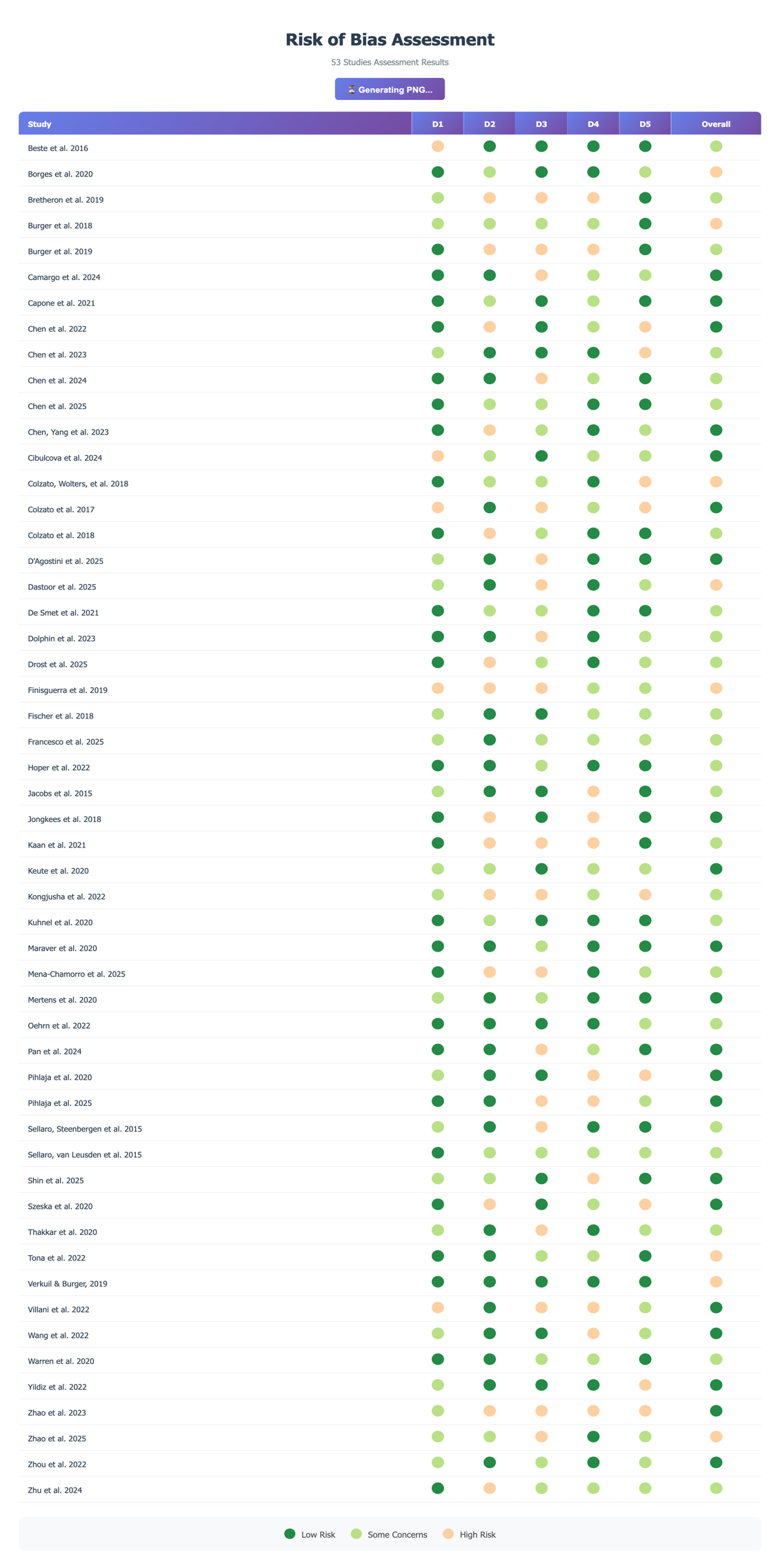


**Figure D1. RoB results traffic-light plot for all included studies.**


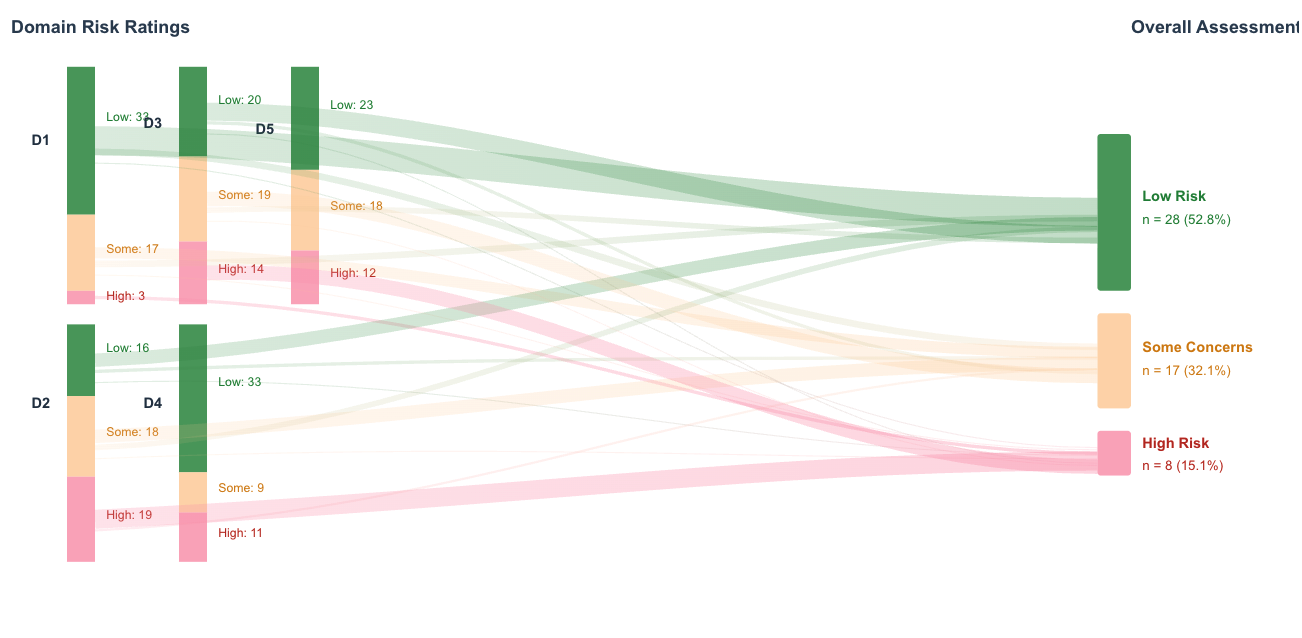


**Figure D2. Sankey diagram linking RoB 2 domain ratings to overall risk classification.**

Note. Overall assessment based on the most unfavourable domain: high risk indicates overall high; all low domains indicate overall low; all others indicate some concern.
