# Supplementary material for: Sorting the mind: cognitive enhancement through transcutaneous auricular vagus nerve stimulation: a systematic review and meta-analysis
Source: Psychol Med. 2026 Jun 24;56:e207. doi: 10.1017/S0033291726105017 (PMC13319486; doi:10.1017/S0033291726105017)
Supplement: Liu and Li supplementary material [file S0033291726105017sup001.zip › PM_Appendix E_Figure E1.docx]

**
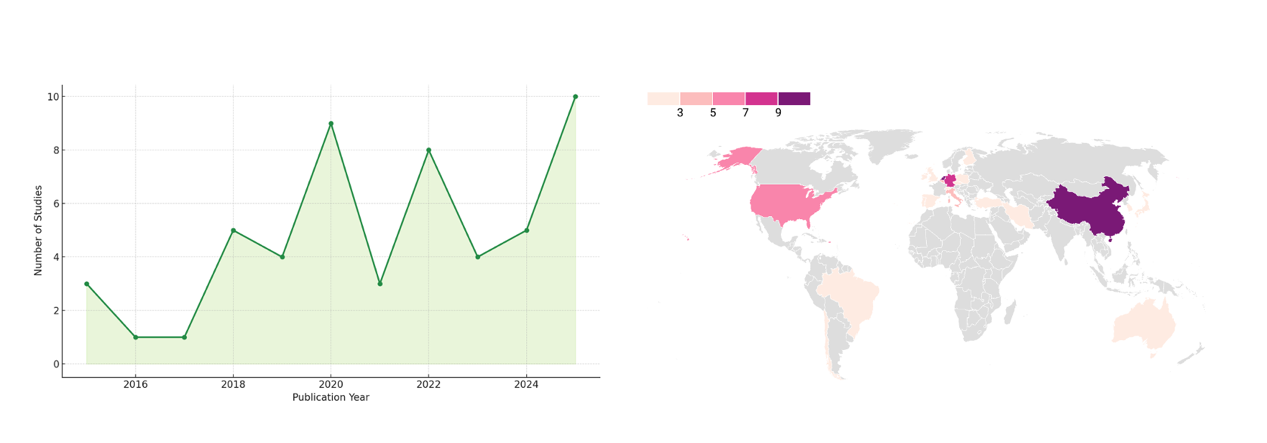
**

**Figure E1. Global and Temporal Distribution of Included taVNS–Cognition Studies
*Note.*** The left panel illustrates the annual publication trend of taVNS studies examining cognitive outcomes from 2015 to 2025, showing a steady rise in output with a marked increase in recent years. The right panel presents a global heatmap of study distribution by primary affiliation, highlighting major research activity in China, Germany, the United States, and Western Europe. Colour intensity reflects the number of studies contributed per country.
