# Supplementary material for: Sorting the mind: cognitive enhancement through transcutaneous auricular vagus nerve stimulation: a systematic review and meta-analysis
Source: Psychol Med. 2026 Jun 24;56:e207. doi: 10.1017/S0033291726105017 (PMC13319486; doi:10.1017/S0033291726105017)
Supplement: Liu and Li supplementary material [file S0033291726105017sup001.zip › PM_Appendix F_Figure F1-5.docx]

**
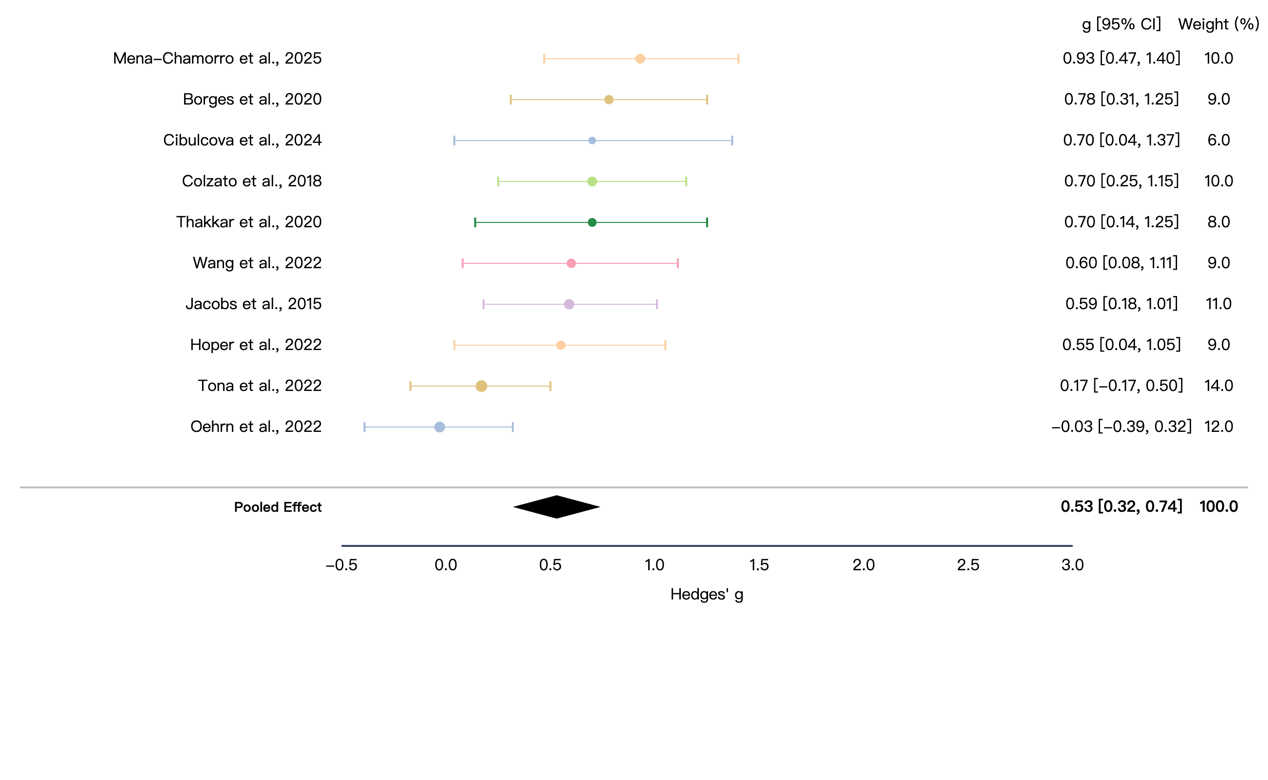
**

**Figure F1. Forest Plot of Cognitive Flexibility and Learning Subgroup.**


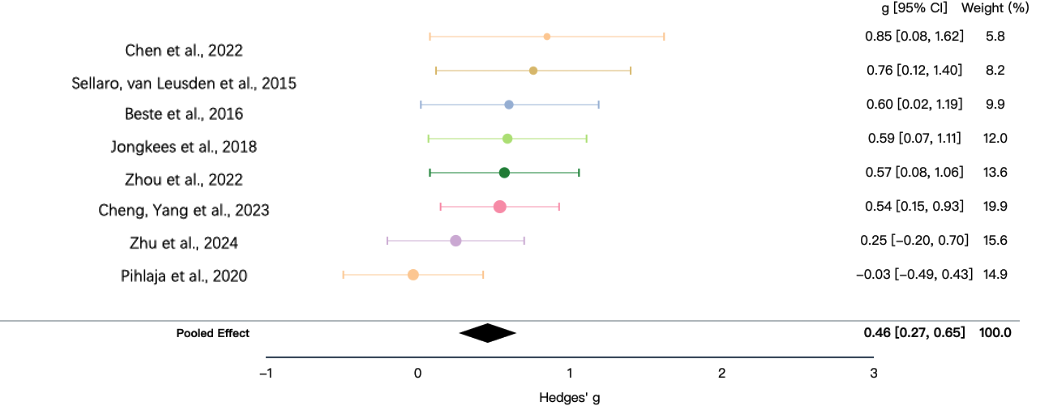


**Figure F2. Forest Plot of Executive Functions Subgroup.**

**
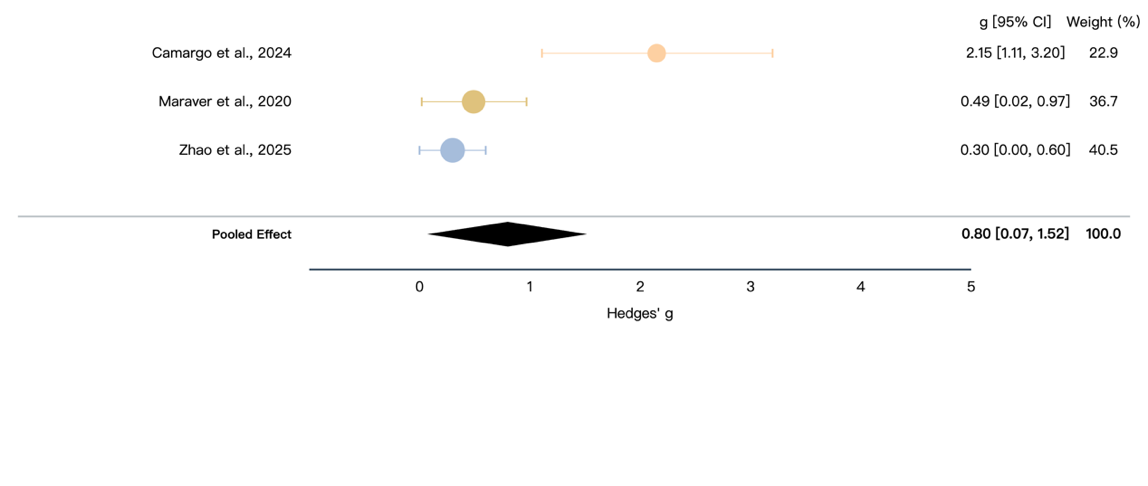
**

**Figure F3. Forest Plot of Social Cognition and Emotion Regulation Subgroup.**

**
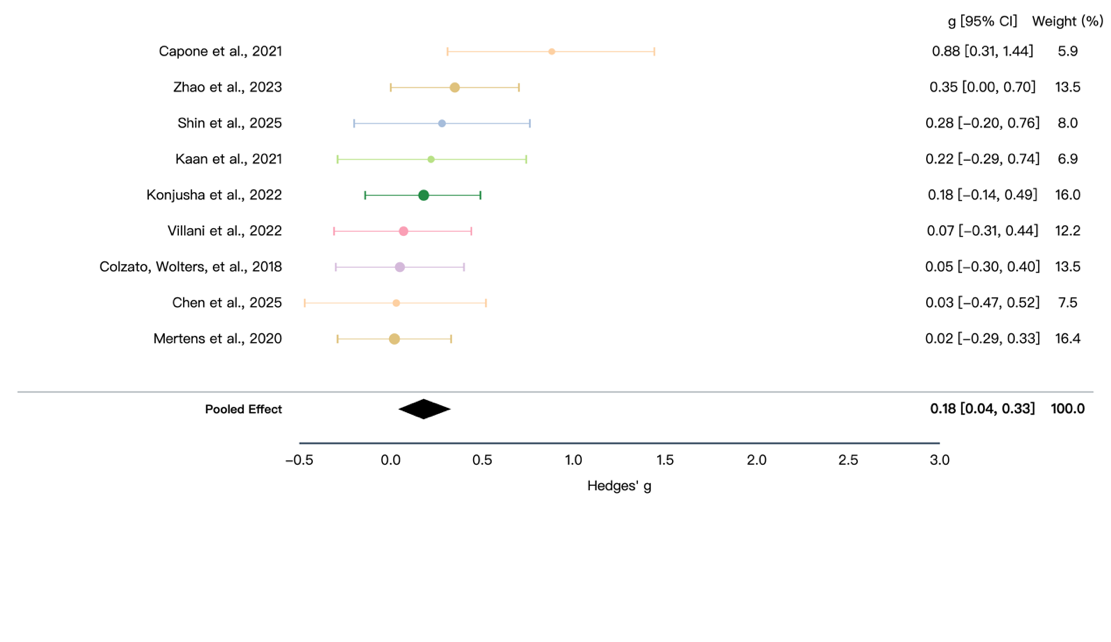
**

**Figure F4. Forest Plot of Working Memory and Attention Subgroup.**


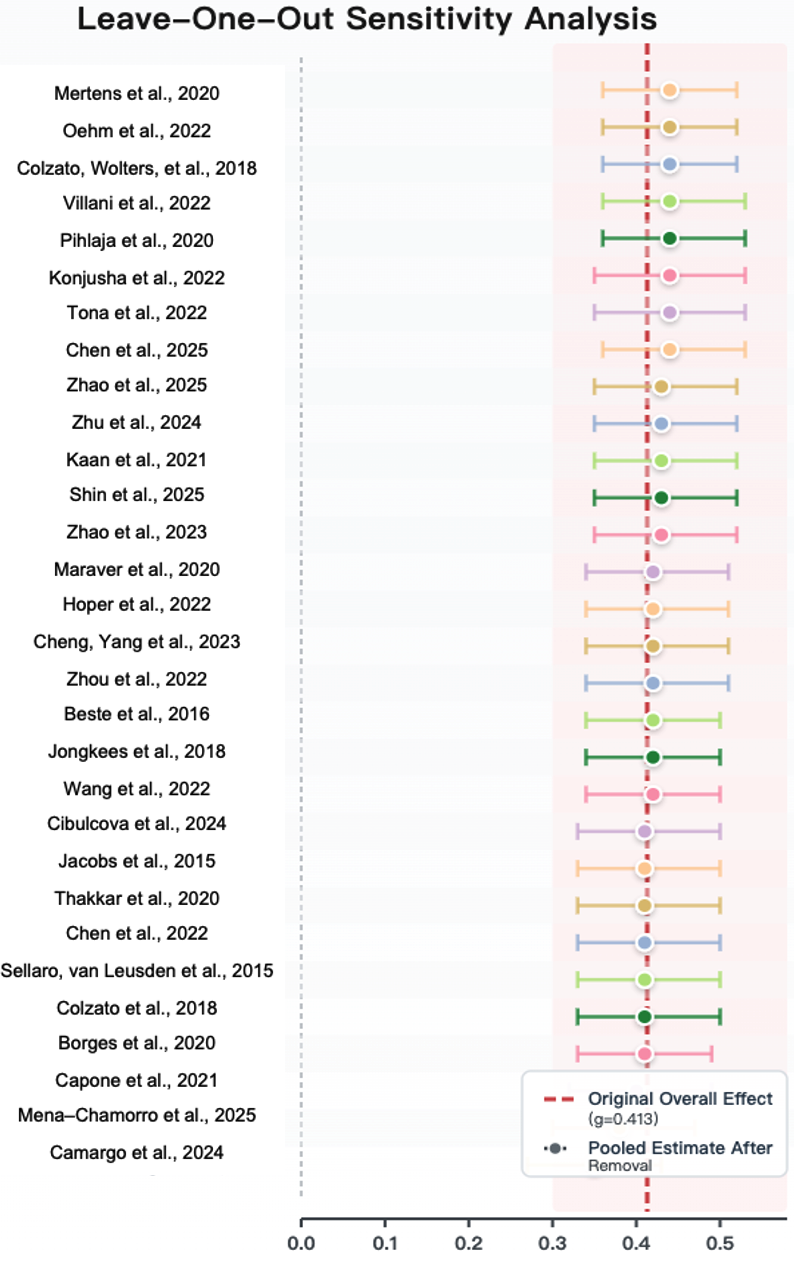


**Figure F5. Leave- One- Out Sensitivity Analysis Results.**

**
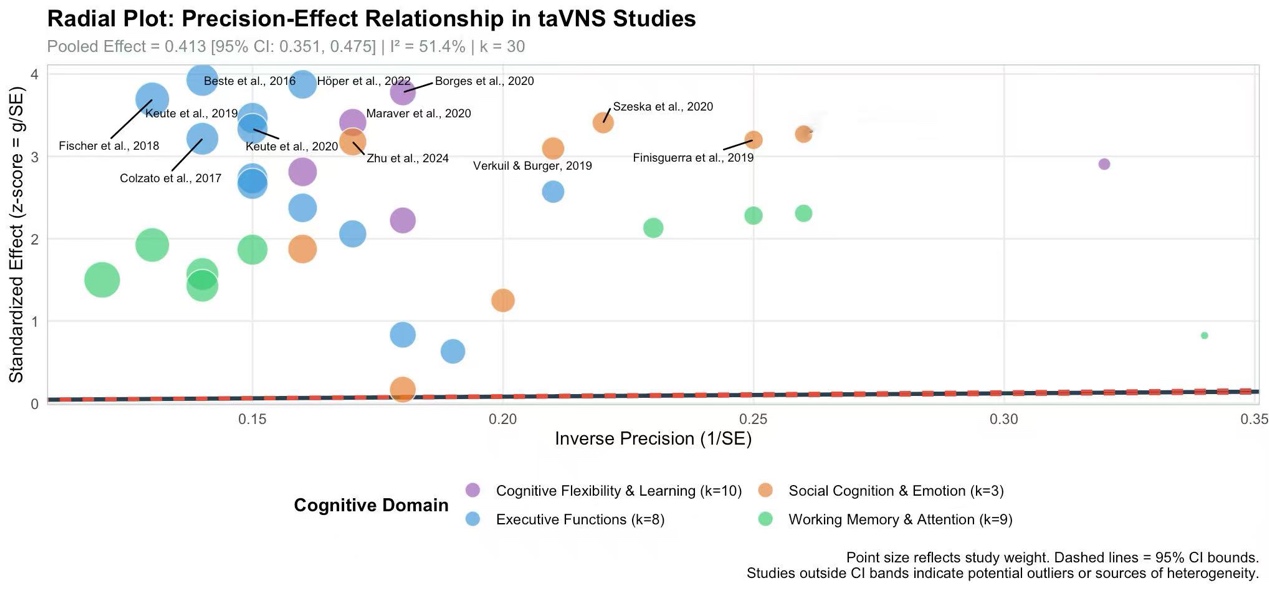
**

**Figure F5. Galbraith (radial) diagram: Standardised effect (g/SE) versus inverse precision (1/SE).**

***Note.*** To avoid clutter, only studies with high impact, lying outside the 95% confidence interval, or being representative are labelled; the remaining studies are not labelled but are all included in the analysis.


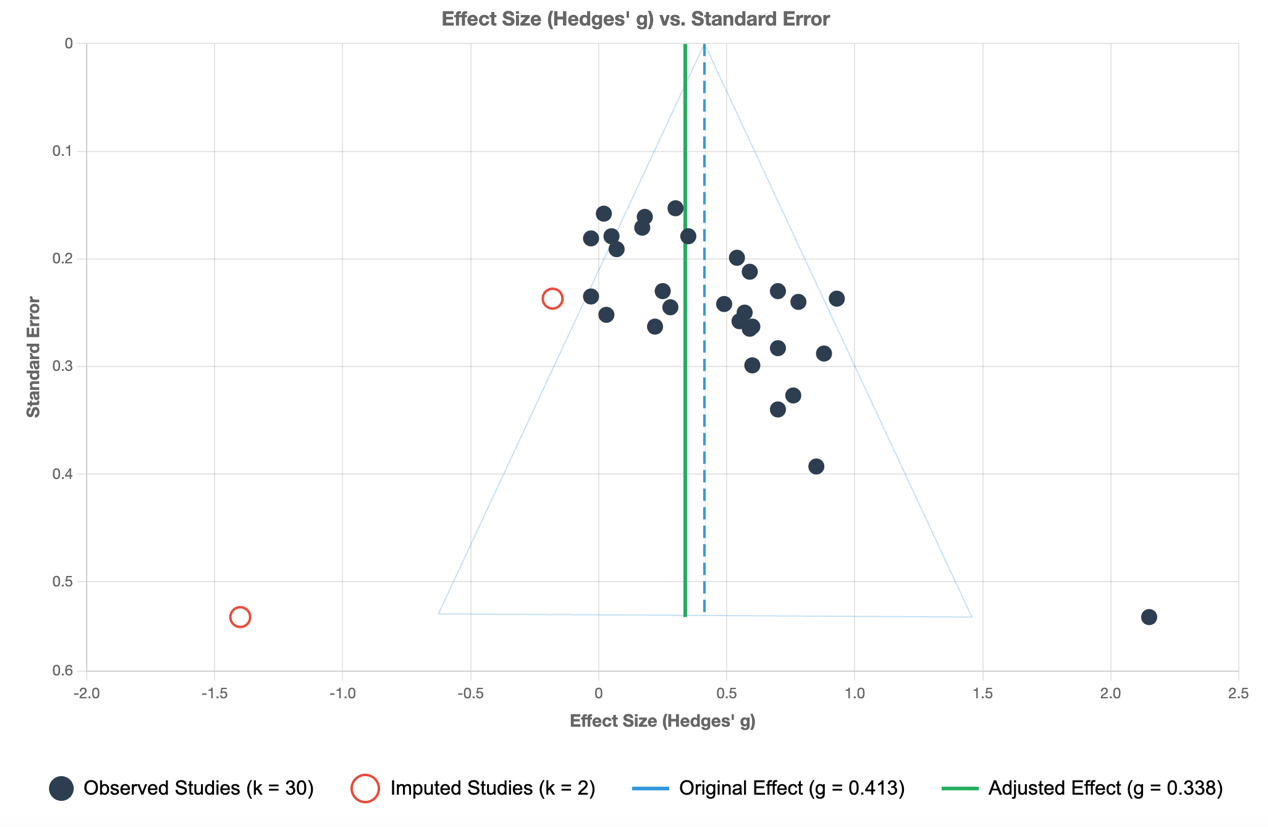


**Figure F6. Funnel Plot for Publication Bias Assessment.**

**Table F1. Publication Bias Analyses Results.**

| **Method** | **Result** | **Interpretation** |
| --- | --- | --- |
| **Egger’s regression test** |  |  |
| Intercept | 3.91 (SE = 1.02) | Significant asymmetry |
| Test statistic | t(28) = 3.83 |  |
| p-value | < .001 | Evidence of bias |
| **Trim-and-fill analysis** |  |  |
| Estimated missing studies | k₀ = 2 (6.7%) | Modest number |
| Studies trimmed | Camargo et al. (2024); Mena-Chamorro et al. (2025) | Extreme positive deviations |
| Original effect size | g = 0.413 [0.295, 0.531] | Before adjustment |
| Adjusted effect size | g = 0.338 [0.220, 0.455] | After adjustment |
| Magnitude of change | −18.2% | Modest reduction |
| Adjusted significance | z = 5.62, p < .001 | Remains significant |
| **Rosenthal’s fail-safe N** |  |  |
| Nfs | 1,067 | High robustness |
| Criterion (5k + 10) | 160 |  |
| Ratio | 6.7:1 | Far exceeds criterion |
| **Orwin’s fail-safe N** |  |  |
| Nfs (criterion g = 0.10) | 94 | Moderate robustness |
| Current sample size | k = 30 |  |
| Ratio | 3.1:1 |  |
