# Supplementary material for: Sorting the mind: cognitive enhancement through transcutaneous auricular vagus nerve stimulation: a systematic review and meta-analysis
Source: Psychol Med. 2026 Jun 24;56:e207. doi: 10.1017/S0033291726105017 (PMC13319486; doi:10.1017/S0033291726105017)
Supplement: Liu and Li supplementary material [file S0033291726105017sup001.zip › PM_Appendix G_ DL and REML Comparison .docx]

**DL and REML Comparison Results**

To test method robustness, DL estimation was used for re-pooling, yielding highly consistent results with REML: the effect size differences (Δg) across domains were small for both methods (e.g., overall cognition Δg = 0.024; executive function Δg = −0.009; social cognition/emotional regulation Δg = 0.147; working memory/attention Δg = 0.014; cognitive flexibility/learning Δg = 0.032). Correlation between methods reached r = .978, 95% CI [.920, .997], r² = .957, p < .001. Bland–Altman analysis revealed a mean difference of 0.0416, with 95% consistency limits [−0.0778, 0.1610], indicating minimal systematic bias.

According to pre-specified protocol criteria, 80% (4/5) domains achieved “excellent agreement”, with the remaining one (“social cognition/emotional regulation”) classified as “moderate agreement”. The latter discrepancy aligns with the subdomain’s limited sample size and high heterogeneity. Importantly, conclusive statistical significance persisted across domains under both REML and DL estimators (p < .05 for both methods), preserving clinical and scientific interpretability.

Pearson correlation between REML and DL estimates was r = .978, 95% CI [.920, .997], r² = .957, indicating 95.7% shared variance and excellent concordance. Bland–Altman analysis showed a mean bias of 0.042 (SD = 0.061) with 95% limits of agreement from −0.078 to 0.161, reflecting minimal systematic bias. Across five cognitive domains, 80% demonstrated “excellent” agreement (Δ < 0.05) and 20% “moderate” agreement (Δ 0.10–0.20); no domain showed poor convergence.

**Table G1**

**Comparison of Effect Sizes Between REML and DerSimonian–Laird (DL) Estimators**

| **Domain** | ***k*** | **REML *g* [95% CI]** | **DL *g*** | **Δ*g*** |
| --- | --- | --- | --- | --- |
| Overall Cognition | 30 | 0.410 [0.300, 0.530] | 0.386 | 0.024 |
| Executive Functions | 8 | 0.462 [0.272, 0.653] | 0.471 | −0.009 |
| Social Cognition | 3 | 0.795 [0.071, 1.519] | 0.648 | 0.147 |
| Working Memory | 9 | 0.185 [0.043, 0.327] | 0.171 | 0.014 |
| Cognitive Flexibility | 10 | 0.534 [0.324, 0.745] | 0.502 | 0.032 |

Note. Δg = difference between REML and DL estimates.

**Table G2**

**Agreement Statistics Between Estimators**

| **Domain** | **% Difference** | **Agreement Status** | **Interpretation** |
| --- | --- | --- | --- |
| Overall Cognition | 5.9% | Excellent | Robust estimate |
| Executive Functions | 1.9% | Excellent | Near-perfect match |
| Social Cognition | 18.5% | Moderate | Expected (small *k*, I² = 82%) |
| Working Memory | 7.6% | Excellent | Stable estimate |
| Cognitive Flexibility | 6.0% | Excellent | Good concordance |

*Note.* Agreement classification based on absolute Δ*g*: Excellent (< 0.05), Good (0.05–0.10), Moderate (0.10–0.20), Poor (> 0.20).


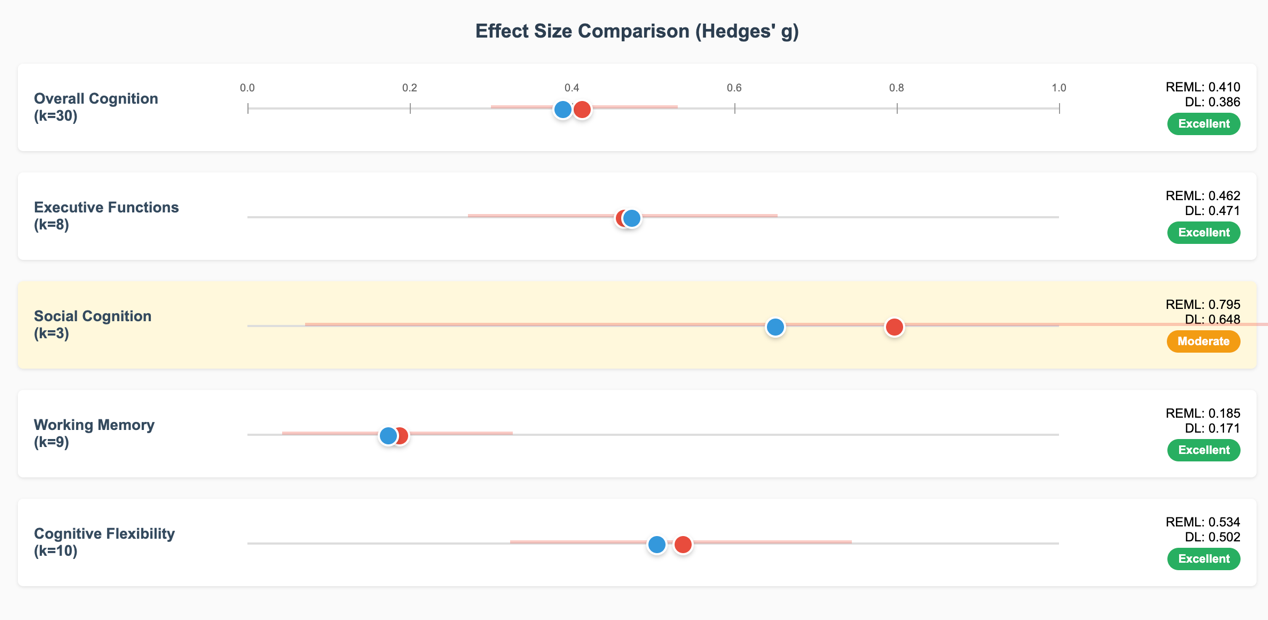


**Figure G1. Effect Sizes Comparison Between DL and REML.**
