# Supplementary material for: Sorting the mind: cognitive enhancement through transcutaneous auricular vagus nerve stimulation: a systematic review and meta-analysis
Source: Psychol Med. 2026 Jun 24;56:e207. doi: 10.1017/S0033291726105017 (PMC13319486; doi:10.1017/S0033291726105017)
Supplement: Liu and Li supplementary material [file S0033291726105017sup001.zip › PM_Appendix H_ Bayesian Results Table H1-2.docx]

**Table H1.**

**taVNS Bayes Meta Summary**

| **Cognitive Domain** | **k** | **Posterior mu (mean)** | **mu 95% CrI [2.5,97.5]** | **Posterior tau (mean)** | **tau 95% CrI [2.5,97.5]** |
| --- | --- | --- | --- | --- | --- |
| Overall | 30 | 0.3956858 | [0.271, 0.531] | 0.25266978 | [0.121, 0.404] |
| Executive Functions | 8 | 0.464118428 | [0.234, 0.702] | 0.166706868 | [0.007, 0.473] |
| Working Memory and Attention | 9 | 0.133355815 | [-0.052, 0.326] | 0.16327178 | [0.007, 0.443] |
| Social Cognition and Emotional Regulation | 3 | 0.626172254 | [-0.321, 1.519] | 0.742950558 | [0.062, 2.081] |
| Cognitive Flexibility and Learning | 10 | 0.529946737 | [0.318, 0.757] | 0.232037279 | [0.033, 0.488] |

**Table H2.**

**taVNS Posterior Forest Data**

| **study_id** | **yi** | **vi** | **posterior_mean** | **posterior_sd** | **CrI_2.5** | **CrI_50** | **CrI_97.5** |
| --- | --- | --- | --- | --- | --- | --- | --- |
| **Beste et al., 2016** | 0.605 | 0.088 | 0.48103971299828500 | 0.19395257238716600 | 0.11265278991922400 | 0.47480071252720500 | 0.875576818573353 |
| **Jongkees et al., 2018** | 0.589 | 0.071 | 0.48062970058570500 | 0.18810065970454400 | 0.12469613275662200 | 0.4746656387537900 | 0.8705144955136100 |
| **Chen et al., 2022** | 0.852 | 0.155 | 0.5262307336706750 | 0.22367572500020400 | 0.11229976188653300 | 0.5148463184514470 | 0.9928493494633660 |
| **Sellaro, van Leusden et al., 2015** | 0.76 | 0.108 | 0.5242858793657120 | 0.20854668394583600 | 0.14636243902309700 | 0.511975682032242 | 0.9580498501861960 |
| **Chen, Yang et al., 2023** | 0.54 | 0.04 | 0.47786240521980900 | 0.15748309700402800 | 0.17631385250078100 | 0.47397870738590700 | 0.794962640263688 |
| **Zhu et al., 2024** | 0.252 | 0.053 | 0.3147046745079900 | 0.16980818431689000 | -0.02476039381270510 | 0.3172583230844520 | 0.6457536233820230 |
| **Pihlaja et al., 2020** | -0.029 | 0.056 | 0.17488464047197100 | 0.1782862498778900 | -0.19741634179190800 | 0.1813660134344620 | 0.5131941774051770 |
| **Capone et al., 2021** | 0.875 | 0.082 | 0.5990436036882160 | 0.20220535933482300 | 0.22905478487029800 | 0.5900252464154820 | 1.0200575969887500 |
| **Konjusha et al., 2022** | 0.178 | 0.026 | 0.24706490271818600 | 0.13526184403545800 | -0.024329356935591100 | 0.248533828093608 | 0.5045408290383310 |
| **Shin et al., 2025** | 0.281 | 0.059 | 0.3392710763790570 | 0.17177059285821700 | 0.0021624112221707700 | 0.33709510013617200 | 0.6841064791399730 |
| **Zhao et al., 2023** | 0.354 | 0.032 | 0.36559694734114300 | 0.14364032473942900 | 0.07735244484668780 | 0.3664575050986240 | 0.6451305570409480 |
| **Colzato, Wolters, et al., 2018** | 0.048 | 0.032 | 0.1748178095165750 | 0.14793252229070700 | -0.1246361874378350 | 0.17845170778237100 | 0.4535856383089640 |
| **Chen et al., 2025** | -0.026 | 0.063 | 0.1877539889392640 | 0.1837081446028060 | -0.19403748534992800 | 0.19178943138324600 | 0.5337889492261820 |
| **Kaan et al., 2021** | -0.222 | 0.069 | 0.10627012451992700 | 0.19535294907176900 | -0.3013072224842550 | 0.11866816879510000 | 0.45852737008492500 |
| **Mertens et al., 2020** | -0.016 | 0.025 | 0.1092652505834690 | 0.1415485512781180 | -0.17462894599048700 | 0.11329721201856600 | 0.37666224269974500 |
| **Villani et al., 2022** | -0.065 | 0.036 | 0.11055571129216400 | 0.15913849288347000 | -0.21494312333486700 | 0.11585167902510500 | 0.4090728573491700 |
| **Camargo et al., 2024** | 2.153 | 0.283 | 0.7196236492293500 | 0.2817460015828500 | 0.2450390390696780 | 0.6954098745726050 | 1.3396106387314500 |
| **Maraver et al., 2020** | 0.493 | 0.059 | 0.4407264676444040 | 0.1756726532186130 | 0.09877799128426140 | 0.4380933894840710 | 0.7953560664706960 |
| **Zhao et al., 2025** | 0.301 | 0.024 | 0.32826000216868500 | 0.13219464222995800 | 0.06458978137680180 | 0.32964662059326800 | 0.5874446776755460 |
| **Borges et al., 2020** | 0.778 | 0.057 | 0.5876229000573290 | 0.18504107989566400 | 0.24795101637364500 | 0.5808948068732840 | 0.9660530575290600 |
| **Colzato et al., 2018** | 0.702 | 0.053 | 0.5536722153822700 | 0.17656014223288600 | 0.22994441549379900 | 0.544328421883921 | 0.9190164207816330 |
| **Cibulcova et al., 2024** | 0.704 | 0.116 | 0.5006341506472030 | 0.20919956329149500 | 0.10827452353024 | 0.49139321342218000 | 0.9284322475541930 |
| **Jacobs et al., 2015** | 0.594 | 0.044 | 0.5040320869618240 | 0.16391662350039800 | 0.19160271020220000 | 0.4998152834620010 | 0.8400731827731460 |
| **Hoper et al., 2022** | 0.547 | 0.067 | 0.46290366770838800 | 0.18265333831153600 | 0.10967416540517500 | 0.4585237345167100 | 0.830970333872713 |
| **Mena-Chamorro et al., 2025** | 0.932 | 0.056 | 0.669514004491868 | 0.19131607209094500 | 0.3227314069733770 | 0.6617042599606280 | 1.0650684538113000 |
| **Thakkar et al., 2020** | 0.697 | 0.08 | 0.5211355323799040 | 0.19470854699142300 | 0.1621410105734640 | 0.5146565059341880 | 0.9251920531524380 |
| **Oehrn et al., 2022** | 0.033 | 0.033 | 0.16850673316671900 | 0.15213568863090100 | -0.13822731549468200 | 0.17300439007016100 | 0.45446108024712300 |
| **Tona et al., 2022** | 0.165 | 0.029 | 0.2437417423059170 | 0.14116525006470300 | -0.04445058565583430 | 0.24635794419890900 | 0.5184106388043760 |
| **Wang et al., 2022** | 0.596 | 0.068 | 0.48661391798560200 | 0.1857602385424070 | 0.1313145567652990 | 0.4802230715669880 | 0.8645247384637000 |
| **Zhou et al., 2022** | 0.568 | 0.062 | 0.4803451880258830 | 0.18059329994947200 | 0.14219107660837300 | 0.4742475772885010 | 0.8534438126430000 |
